# Functional screening of lysosomal storage disorder genes identifies modifiers of alpha-synuclein neurotoxicity

**DOI:** 10.1371/journal.pgen.1010760

**Published:** 2023-05-18

**Authors:** Meigen Yu, Hui Ye, Ruth B. De-Paula, Carl Grant Mangleburg, Timothy Wu, Tom V. Lee, Yarong Li, Duc Duong, Bridget Phillips, Carlos Cruchaga, Genevera I. Allen, Nicholas T. Seyfried, Ismael Al-Ramahi, Juan Botas, Joshua M. Shulman

**Affiliations:** 1 Department of Neuroscience, Baylor College of Medicine, Houston, Texas, United States of America; 2 Department of Neurology, Baylor College of Medicine, Houston, Texas, United States of America; 3 Quantitative and Computational Biology Program, Baylor College of Medicine, Houston, Texas, United States of America; 4 Department of Molecular and Human Genetics, Baylor College of Medicine, Houston, Texas, United States of America; 5 Medical Scientist Training Program, Baylor College of Medicine, Houston, Texas, United States of America; 6 Departments of Biochemistry and Neurology, Emory University School of Medicine, Atlanta, Georgia, United States of America; 7 Department of Psychiatry, Washington University, St. Louis, Missouri, United States of America; 8 NeuroGenomics and Informatics, Washington University, St. Louis, Missouri, United States of America; 9 Departments of Electrical and Computer Engineering, Computer Science, and Statistics, Rice University, Houston, Texas, United States of America; 10 Jan and Dan Duncan Neurological Research Institute, Texas Children’s Hospital, Houston, Texas, United States of America; 11 Center for Alzheimer’s and Neurodegenerative Diseases, Baylor College of Medicine, Houston, Texas, United States of America; Stanford University School of Medicine, UNITED STATES

## Abstract

Heterozygous variants in the *glucocerebrosidase* (*GBA*) gene are common and potent risk factors for Parkinson’s disease (PD). *GBA* also causes the autosomal recessive lysosomal storage disorder (LSD), Gaucher disease, and emerging evidence from human genetics implicates many other LSD genes in PD susceptibility. We have systemically tested 86 conserved fly homologs of 37 human LSD genes for requirements in the aging adult *Drosophila* brain and for potential genetic interactions with neurodegeneration caused by α-synuclein (αSyn), which forms Lewy body pathology in PD. Our screen identifies 15 genetic enhancers of αSyn-induced progressive locomotor dysfunction, including knockdown of fly homologs of *GBA* and other LSD genes with independent support as PD susceptibility factors from human genetics (*SCARB2*, *SMPD1*, *CTSD*, *GNPTAB*, *SLC17A5*). For several genes, results from multiple alleles suggest dose-sensitivity and context-dependent pleiotropy in the presence or absence of αSyn. Homologs of two genes causing cholesterol storage disorders, *Npc1a / NPC1* and *Lip4 / LIPA*, were independently confirmed as loss-of-function enhancers of αSyn-induced retinal degeneration. The enzymes encoded by several modifier genes are upregulated in αSyn transgenic flies, based on unbiased proteomics, revealing a possible, albeit ineffective, compensatory response. Overall, our results reinforce the important role of lysosomal genes in brain health and PD pathogenesis, and implicate several metabolic pathways, including cholesterol homeostasis, in αSyn-mediated neurotoxicity.

## Author summary

Parkinson’s disease (PD) is characterized clinically by progressive decline in both motor and non-motor brain functions, and pathologically by the aggregation of alpha-synuclein protein along with neuron loss. Partial loss-of-function in the *glucocerebrosidase* (*GBA*) gene causes a 5-fold increased risk of PD. By contrast, nearly complete loss of *GBA* causes a distinct disorder called Gaucher’s disease, and other metabolic genes that cause lysosomal storage disorders (LSDs) have been similarly implicated in PD risk. Using a fruit fly model, we have tested 86 conserved homologs of human LSD genes for interactions with alpha-synuclein-induced neurotoxicity. Our screen identified 15 enhancers of progressive locomotor dysfunction, including knockdown of fly homologs of *GBA*, other LSD genes with support from human genetics, as well as new candidate PD genes, such as *LIPA* and *NPC1*, which are involved in cholesterol metabolism. Our results suggest that partial loss of many LSD genes may enhance alpha-synuclein-mediated PD risk and pathogenesis, whereas more complete loss impairs nervous system function independent of alpha-synuclein. We also discover that alpha-synuclein may trigger increases of selected LSD proteins. Overall, this work reinforces the important role of lysosomal metabolism in brain health and PD pathogenesis.

## Introduction

Parkinson’s disease (PD) is a common and incurable neurodegenerative disorder with strong evidence for genetic etiology [1]. Heterozygous carriers for variants in the *glucocerebrosidase* (*GBA*) gene have an approximately 5-fold increased risk of PD, and *GBA* variants also modify PD clinical manifestations, causing more rapid progression and susceptibility for dementia [1,2]. Whereas partial loss-of-function is associated with PD, complete or near-complete loss of *GBA* causes Gaucher disease, a recessive lysosomal storage disorder (LSD) [3,4]. *GBA* encodes the lysosomal enzyme glucocerebrosidase (GCase), which catalyzes the breakdown of glucosylceramide, a substrate that accumulates along with other, more complex sphingolipids in Gaucher disease and possibly PD [5].

There are more than 50 different LSDs, which are similarly characterized by defects in lysosomal biogenesis and/or function, and lead to heterogeneous clinical manifestations, including neurodegeneration in many cases [6]. Emerging evidence from human genetics suggests that other LSD genes, beyond *GBA*, may also influence PD susceptibility. For example, heterozygous carriers of loss-of-function variants in *SMPD1*, which cause Niemann Pick Disease type A/B, have been shown to increase PD risk [7]. Moreover, in independent studies, an aggregate burden of rare, damaging variants in LSD genes was associated with PD, and this relation was robust to exclusion of *GBA* [8,9]. Although the rarity of variants limits statistical power to definitively establish the responsible genes, suggestive evidence implicates possible roles for *CTSD*, *SLC17A5*, and *ASAH1* [8]. Lastly, based on genome-wide association study meta-analysis, common variants implicate several other LSD genes at PD risk loci, including *SCARB2*, *GRN*, *GUSB*, *GALC*, and *NAGLU* [10].

While the precise mechanism by which *GBA* variants affect PD risk remains unknown, substantial evidence points to interactions with α-synuclein protein (αSyn), which aggregates to form Lewy body pathology. αSyn disrupts endolysosomal trafficking, including transport of GCase and other lysosomal enzymes, leading to reduced enzymatic activity and metabolic perturbations [11,12]. Reciprocally, loss of GCase may promote Lewy body pathology due to increased αSyn protein and aggregation [13], resulting from impaired lysosomal autophagy [14] and sphingolipid substrate accumulation [12,15,16]. In this study, we leverage a versatile *Drosophila* model to systematically test the hypothesis that other LSD genes may similarly interact with α-synuclein-mediated neurotoxic mechanisms. Our results highlight requirements for many LSD genes in the maintenance of central nervous system (CNS) structure and function, and further reinforce links with PD pathogenesis.

## Results

### Associations of LSD genes with PD risk

Previously, we and others have discovered evidence for an aggregate burden of rare genetic variants among LSD genes in association with PD risk [8,9]. Although several LSD genes have also been implicated at susceptibility loci from PD genome-wide association studies [10], to our knowledge, a systematic analysis for common variant associations across all LSD genes has not been performed. Leveraging publicly available summary statistics from 56,306 PD cases and 1.4 million control subjects [10], we used the multi-marker analysis of genomic annotation (MAGMA) tool [17] to examine LSD genes for enrichment of variants associated with PD. MAGMA computes an overall gene-set test statistic considering all variants falling within gene intervals, including adjustments for gene size and regional linkage disequilibrium. The full LSD gene set was significantly enriched for variants associated with PD risk (n = 51 loci, p = 0.0011) (S1 Table); the X-linked LSD genes (*GLA*, *IDS*, and *LAMP2*) were excluded from the available genome-wide association dataset. In order to identify possible drivers for the gene set association, we examined MAGMA output considering each of the LSD genes independently. These results identify *GBA* and 9 other LSD genes with aggregate evidence for common variant associations (p < 0.05): *IDUA*, *SCARB2*, *CLN8*, *GNPTAB*, *ARSA*, *GALC*, *CLN5*, *NAGLU*, and *CTSD*. We also performed a sensitivity analysis showing that the LSD gene set association remains significant after excluding either *GBA* (n = 50 loci, p = 0.014) or the top 3 genes (*GBA* plus *SCARB2* and *IDUA*) (n = 47 loci, p = 0.03), which are similarly localized to regions with genome-wide significant associations in the dataset. These results reinforce the genetic connection between causes of LSDs and PD, revealing an important role for common variant associations.

### Screen for LSD gene modifiers of α-synuclein-mediated neurotoxicity

**Fig 1:**
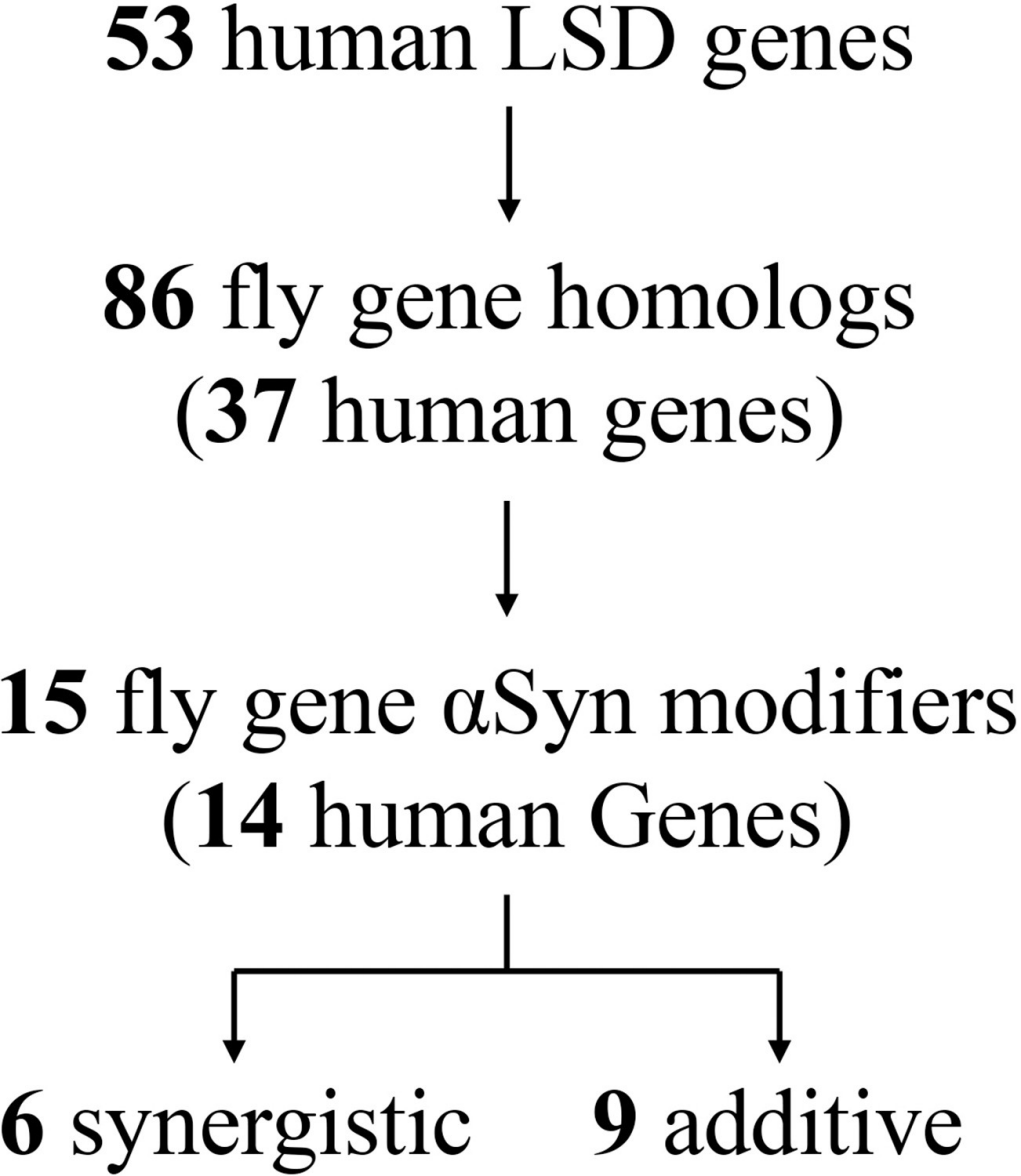
Study flowchart. Out of 53 human LSD genes, 86 are conserved in *Drosophila* and have available lines for genetic screening. 259 genetic fly strains, including RNAi and insertional or classical alleles, were tested for interaction with the locomotor phenotype induced by pan-neuronal α-synuclein (αSyn) expression. Screening revealed 15 fly genes for which gene loss significantly enhances the αSyn phenotype. Based on further tests of all modifying strains in the absence of αSyn, genes were further classified as synergistic or additive. Synergistic genes were defined as having *at least one* modifying allele in which there was no significant evidence of locomotor impairment in the absence of αSyn.

Due to the rarity of pathogenic variants, human genetic studies are underpowered to comprehensively resolve all of the LSD genes contributing to PD risk and pathogenesis [8]. As a complementary approach, and to systematically test the hypothesis that LSD genes may broadly interact with αSyn-mediated neurotoxicity, we implemented a cross-species strategy. Pan-neuronal expression of the human *SNCA* gene in the fruit fly, *Drosophila melanogaster*, causes Lewy body-like αSyn aggregates, lysosomal stress, dopaminergic and other neuronal loss, and progressive locomotor impairment [18,19]. We therefore performed a genetic modifier screen examining for interactions between homologs of human LSD genes and αSyn-induced neurotoxicity, using locomotor behavior as a readout for CNS function (Fig 1). Out of 53 human LSD genes [8], 39 (74%) are conserved in flies (S2 Table). Overall, our screen considered 86 homologs (many genes had multiple conserved homologs), and we obtained 259 distinct strains for genetic manipulation, including RNA interference (RNAi) and other available alleles (mean of ~3 independent experimental manipulations per gene; S3 Table). We employed an automated locomotor behavioral assay based on the *Drosophila* negative geotactic response, which is highly amenable for high-throughput genetic screening [20,21].

**Fig 2:**
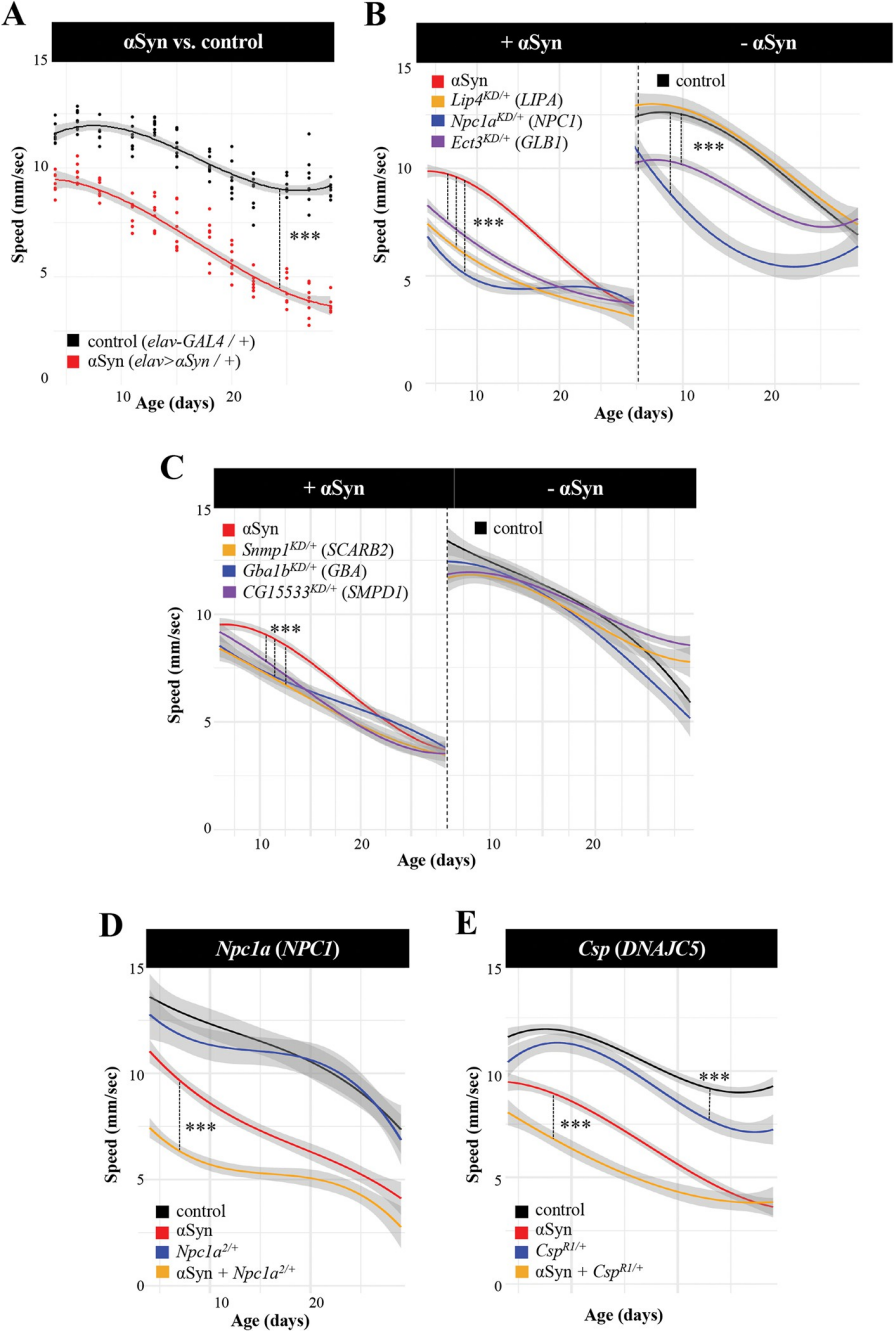
Lysosomal storage disorder (LSD) gene homologs show dose-sensitivity and context-dependent pleiotropy in *Drosophila*. (A) Pan-neuronal expression of human α-synuclein (*elav > αSyn*) induces progressive locomotor impairment. (B) Pan-neuronal knockdown (KD) of LSD gene homologs with RNA-interference (RNAi) enhances the αSyn locomotor phenotype. RNAi transgenes were tested in heterozygosity. In the absence of αSyn, KD (*elav >* RNAi) causes no (*LIPA/Lip4*: *v31021*), moderate (*GLB1/Ect3*: *3132R1*), or severe (*NPC1/Npc1a*: *v105405*) toxicity. (C) Additional synergistic gene modifiers enhance αSyn following KD, but do not cause significant locomotor impairment in the absence of αSyn (*SCARB2/Snmp1*: *v42496; GBA/Gba1b*: *v21336;* and *SMPD1/CG15533*: *v42520*). (D and E) Heterozygous loss-of-function alleles of *Npc1a* (D) and *Csp* (E) dominantly enhance αSyn. Climbing speed was assessed longitudinally including at least 11 aged time points over 30 days (n > 6 replicates of 15 animals each). Statistical comparisons based on one-way ANOVA considering three nested models (genotype, genotype + time, and genotype*time) and reporting results for the most complex model meeting significance. ***, p<5x10^-5^. See also S1 Fig for comprehensive results from validation tests of all other genes/alleles, and S5 Table for detailed statistical output.

**Fig 3:**
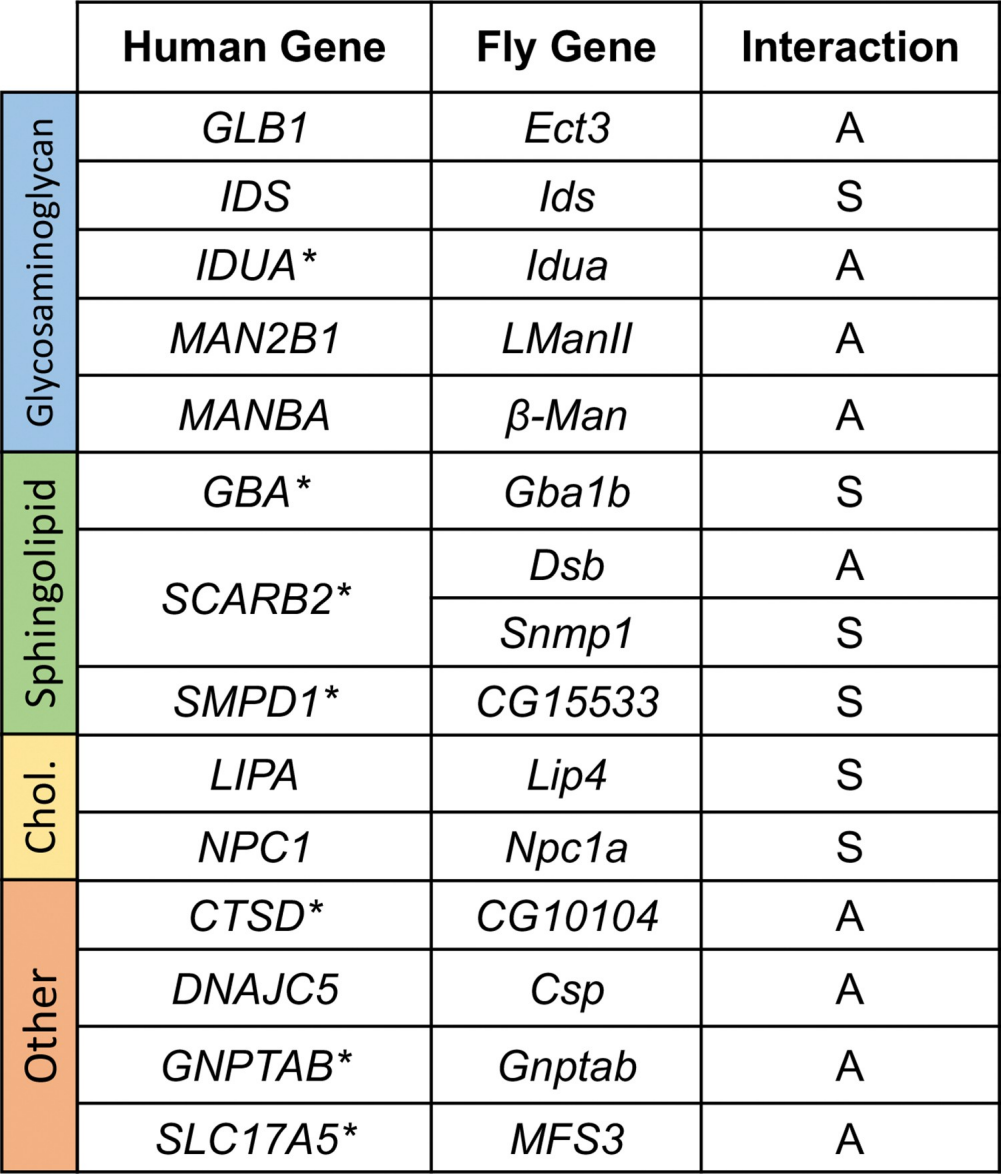
Lysosomal storage disorder (LSD) gene modifiers of α-synuclein (αSyn). Human LSD genes and *Drosophila* homologs identified as modifiers are indicated. Human genes with additional supportive evidence as PD risk loci from human genetics are noted with an asterisk (*). Based on further tests to establish αSyn-dependent or independent activity, genes were further classified as synergistic (S) or additive (A). Synergistic genes were defined as having at least one modifying allele in which there was no significant evidence of locomotor impairment in the absence of αSyn. Additive genes were characterized by alleles that consistently produced locomotor phenotypes in the absence of αSyn. All modifier genes had at least one allele establishing αSyn-independent functional requirements in the fly nervous system.

Compared to controls (*elav-GAL4 / +*), pan-neuronal expression of human αSyn (*elav> αSyn / +*) causes progressive locomotor impairment (Fig 2A). RNAi transgenes targeting the fly homologs of human LSD genes were coexpressed throughout the nervous system with *αSyn* using the same *elav-GAL4* driver, and climbing speed of adult flies was evaluated longitudinally between 1 and 3 weeks of age. All transgenes were tested in heterozygosity. *Drosophila* RNAi lines are designed for optimal specificity [22,23]. To further minimize the possibility of off-target effects, we only considered genes as modifiers when supported by consistent evidence from at least two independent RNAi strains or other alleles. Overall, our screen identified 15 fly genetic modifiers of αSyn, homologous to 14 human LSD genes (two different paralogs of *SCARB2* were identified as modifiers) (Fig 3). In all cases, genetic manipulations predicted to reduce the function of LSD gene homologs (RNAi knockdown or other loss-of-function alleles) enhanced the *elav>αSyn* locomotor phenotype (Figs 2B–2E and S1).

Since LSDs are frequently associated with neurologic manifestations, we reasoned that knockdown of *Drosophila* homologs would likely cause CNS dysfunction in many cases, independent of αSyn expression. Therefore, we also tested all modifying lines from our screen (35 RNAi and other alleles) to examine the consequences for locomotor behavior in the absence of αSyn (e.g., *elav>RNAi / +* versus *elav-GAL4 / +*). These experiments revealed a range of phenotypic severity (Figs 2B and S1), with some RNAi lines showing little or no locomotor phenotype (e.g., *LIP4 / Lip4*) and others with mild (e.g., *GLB1 / Ect3*) or more substantial, age-dependent impairments (e.g., *NPC1 / Npc1a*). Based on these results, we classified the 15 genes as either “additive” or “synergistic” modifiers of αSyn (Fig 3). Synergistic modifiers had at least one modifying allele in which there was no significant evidence of locomotor impairment in the absence of αSyn (Fig 2C). Overall, 6 of 15 genes, including *Gba1b*, the *Drosophila* homolog of *GBA*, showed evidence of synergistic interactions with αSyn mediated neurotoxicity. The remaining “additive” genetic modifiers were characterized by alleles that consistently produced locomotor phenotypes in the absence of αSyn. Notably, all LSD modifier genes had at least one allele tested revealing αSyn-independent functional requirements in the aging nervous system.

In humans, *GBA* reveals dose-sensitive and pleiotropic effects on disease traits, causing Gaucher disease and also increasing susceptibility for PD. In *Drosophila*, our screen results also suggest that many LSD gene homologs may have similar dose-dependent relationships (S1 Fig). For 3 loci, including *GBA* (*Gba1b*), *IDS* (*Ids*), and *LIPA* (*Lip4*), knockdown with multiple, independent RNAi transgenes targeting each of these genes showed consistent enhancement of αSyn-induced locomotor impairment, but differential requirements in the absence of αSyn. For selected genes, classical mutant alleles were also available and permitted evaluation of potential heterozygous interactions. Strikingly, heterozygous loss-of-function alleles for both *Npc1a* and *Csp*, homologous to human *NPC1* and *DNAJC5*, respectively, dominantly enhanced αSyn, but caused little to no phenotype when examined on their own (Figs 2D, 2E and S1). By contrast, RNAi-knockdown of both genes induced a marked locomotor phenotype independent of αSyn (Figs 2B and S1). Overall, our results are potentially consistent with a model in which partial loss of function for multiple LSD genes may enhance αSyn neuropathology, as with *GBA*-PD, but that more complete loss of gene function may compromise CNS function, as in neuronopathic Gaucher disease and many other LSDs. For several genes of interest, we confirmed that both RNAi and heterozygous loss-of-function alleles caused decreased gene expression; however, we were not able to establish a predictable relationship between degree of knockdown and severity of locomotor phenotype, at least based solely on mRNA levels (S2 Fig; see also Discussion).

### Cholesterol metabolism and α-synuclein mediated neurotoxicity

LSDs are commonly classified based on the metabolic pathways disrupted and characteristic type of substrate accumulation. For example, *GBA*, *SCARB2*, and *SMPD1*, which are collectively implicated in PD risk, are also jointly involved in sphingolipid metabolism; fly homologs of all 3 genes (*Gba1b*, *Dsb*, and *CG15533*, respectively) were identified in our screen as synergistic, loss-of-function enhancers of αSyn. Among our results, *Npc1a* and *Lip4* are both fly homologs of genes causing the human cholesterol storage disorders, Niemann Pick Disease type C (*NPC1*) and cholesterol ester storage disease (*LIPA*). Both *Npc1a* and *Lip4* were notable for robust, synergistic enhancement following gene knockdown or in the presence of heterozygous mutant alleles, consistent with dose-sensitive interactions with αSyn-mediated neuronal injury (Fig 2B and 2D). Notably, pan-neuronal overexpression of either gene using available lines did not suppress but rather mildly enhanced the αSyn locomotor phenotype, consistent with a one-way interaction (S3 Fig; see also Discussion). Whereas *Lip4* has not been well-characterized in flies, loss of *Npc1a* causes the accumulation of cholesterol, defective synaptic transmission, and ultimately, neurodegeneration, similar to human Nieman Pick type C [24]. We confirmed significant, albeit modest, elevations of total cholesterol levels in fly heads following genetic manipulations of either *Npc1a* or *Lip4* (S4 Fig).

The lysosome has been implicated as an important regulator of αSyn proteostasis and toxicity. In order to begin to address potential mechanisms by which loss-of-function in cholesterol storage disorder genes might enhance αSyn-mediated neuronal injury, we therefore examined two well-established markers of lysosomal function [25]: the autophagy mediator, p62, and Cathepsin L, which is cleaved to generate a mature enzyme. However, following genetic manipulations of *Npc1a* or *Lip4*, we did not detect any changes in either of these markers to suggest a global lysosomal dysfunction (S5 and S6 Figs). Moreover, using a sensitive ELISA assay, we confirmed that levels of total αSyn protein were largely stable following manipulations of *Npc1a* or *Lip4* as well as all other LSD gene modifiers identified in our screen (S7 Fig).

**Fig 4:**
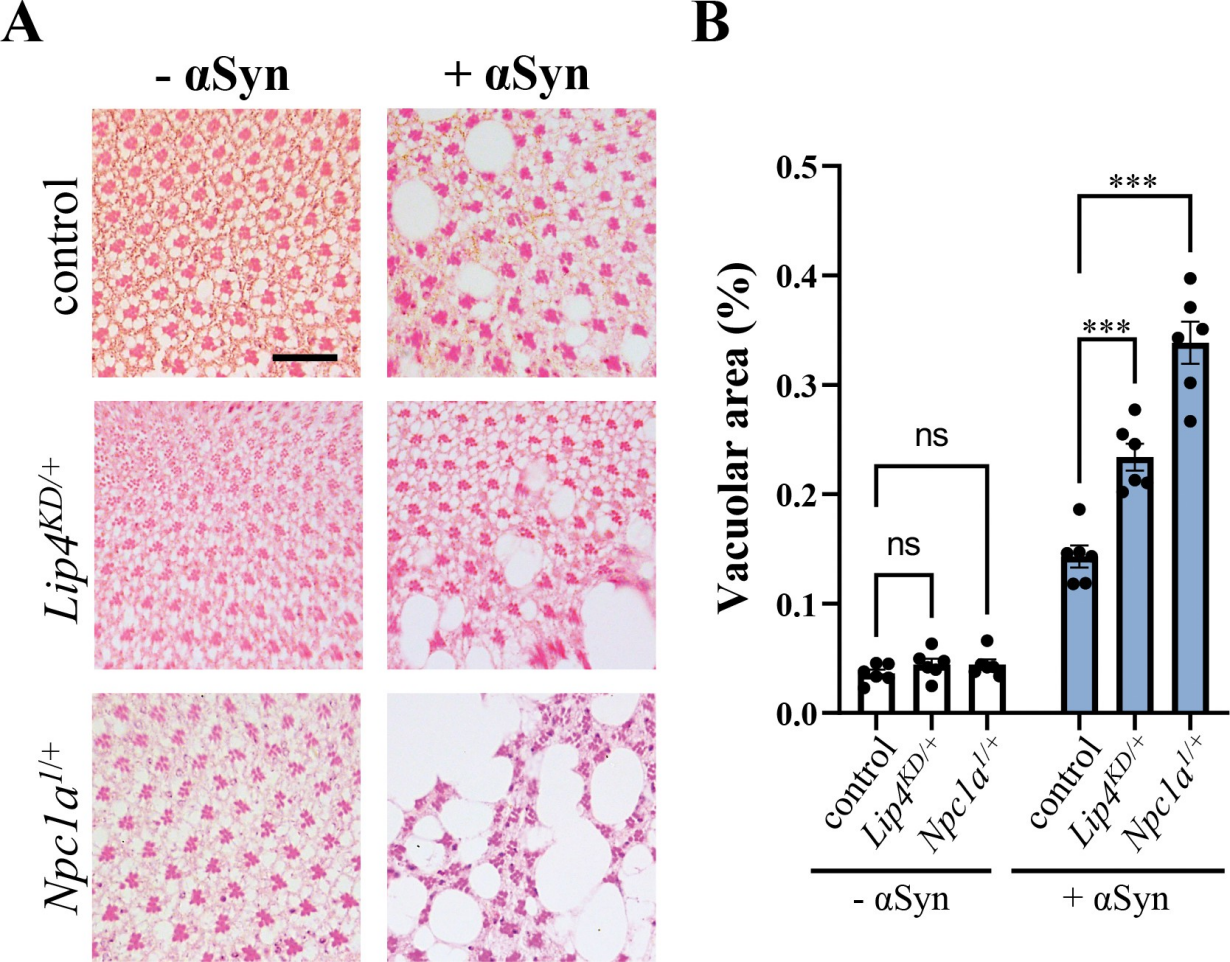
Cholesterol storage disorder gene homologs enhance α-synuclein (αSyn) induced retinal degeneration. (A) Expression of αSyn in the adult retina (*Rh1>αSyn*) causes progressive neurodegeneration compared with controls (*Rh1-GAL4 / +*). Knockdown (KD) of *Lip4* (*Rh1>v31021 / +*) or heterozygosity for a *Npc1a* loss-of-function allele enhances αSyn-mediated tissue destruction. Tangential retinal sections from 15 day-old animals were stained with hematoxylin and eosin. (B) Quantification based on extent of vacuolar changes (vacuole area / total area) from at least n = 6 animals per genotype. Statistical comparisons were made using unpaired t-tests, followed by Dunnett’s post-hoc test. Error bars represent the standard error of the mean. ***, p<0.001; ns, not significant. Scale bar = 20μm. See also S8 Fig for results using additional RNAi and alleles.

In order to confirm the interactions between αSyn and *Npc1a* and *Lip4*, we used an independent retinal neurodegeneration assay. We previously established that expression of αSyn in adult photoreceptors using the *Rh1-GAL4* driver (*Rh1>* α*Syn*) causes age-dependent structural degeneration [25], with mild vacuolar changes manifested at 15 days, based on hematoxylin and eosin staining of tangential sections through the retina (Figs 4 and S8). RNAi-mediated *Lip4* knockdown or a heterozygous *Npc1a* loss-of-function allele significantly increased αSyn-induced retinal degeneration, but similar changes were not seen in corresponding controls. These results are consistent with the findings from our screen and further implicate lysosomal regulators of cholesterol metabolism in αSyn-mediated neurotoxicity.

### α-synuclein pathology causes altered expression of LSD enzymes

**Fig 5:**
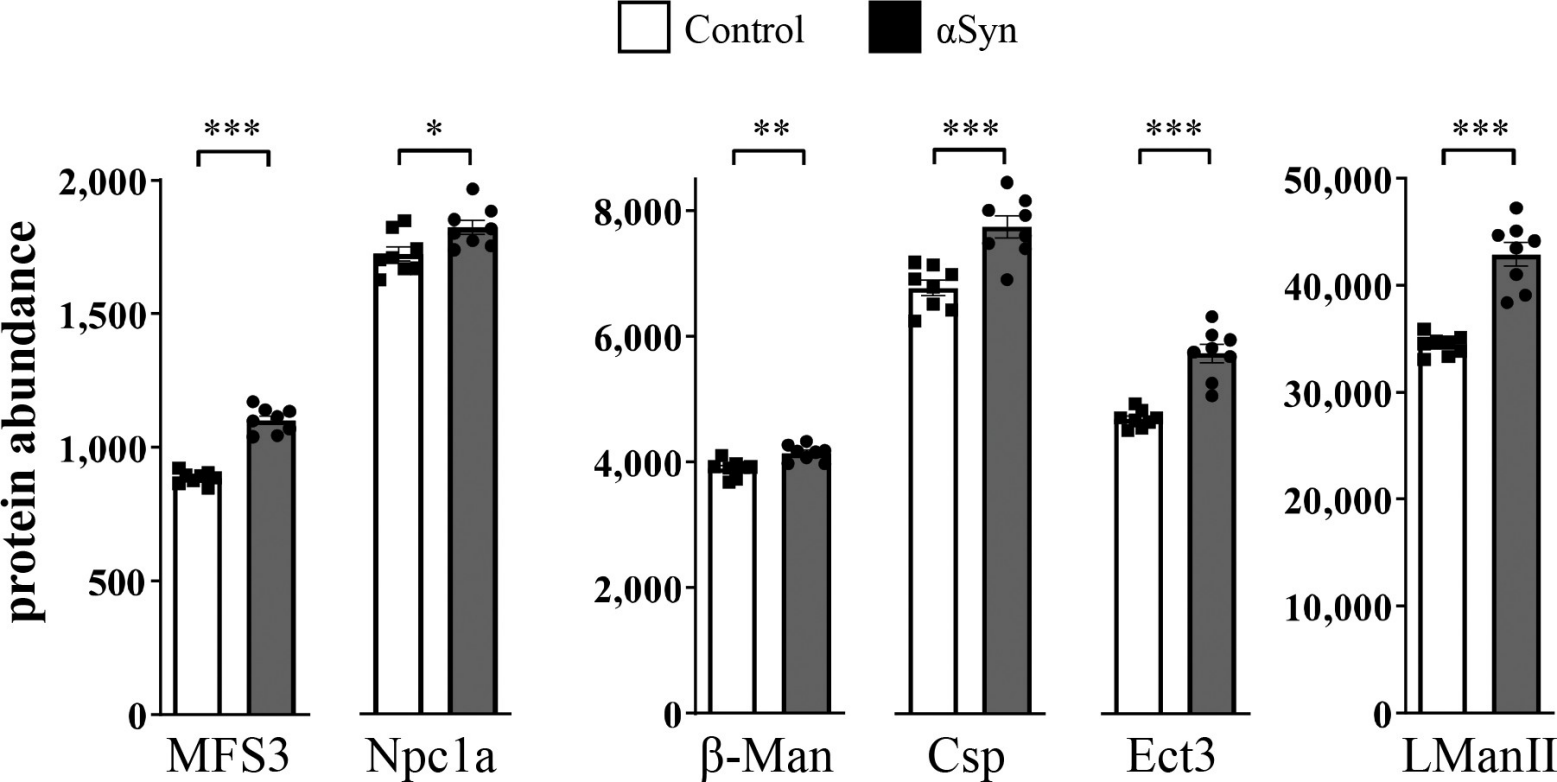
Differential expression of lysosomal storage disorder (LSD) protein homologs in flies. Comparisons of protein abundance (normalized) from fly heads are shown for *elav>αSyn* (Gray) or control (White, *elav-GAL4 / +*), based on Tandem Mass Tag proteomics. t-tests were performed for comparisons of mean abundance considering n = 8 replicate samples for each genotype. Error bars represent the standard error of the mean. *, p<0.05; **, p<0.01; ***, p<0.001. All proteins shown were also significantly differentially expressed based on analyses in DESeq2 (Wald test) and following adjustment using the Benjamini-Hochberg procedure (p_adj_ < 0.05). See also S4 Table and S9 Fig for replication analysis using an independent longitudinal *Drosophila* proteomics dataset.

To further explore underlying mechanisms, we generated unbiased mass-spectrometry proteomics from αSyn (*elav>αSyn / +*) and control (*elav-GAL4 / +*) animals, using the same genotypes as in our locomotor screen and initially choosing a timepoint (10-days) that is predicted to be early in the overall pathologic progression. From these data, 48 *Drosophila* homologs of human LSD proteins were detected. Strikingly, 22 fly proteins (homologous to 16 human proteins encoded by LSD genes) were significantly differentially expressed following pan-neuronal expression of αSyn in the adult brain (Fig 5 and S4 Table), including 15 up- and 7 down-regulated proteins. Importantly, many proteins overlapped with genetic modifiers identified in our screen. In an independent longitudinal proteomics dataset we replicated αSyn-induced increases among 6 of these proteins (S9 Fig), including Npc1a and several proteins homologous to human LSD gene products causing mucopolysaccharidoses: GLB1/Ect3, MAN2B1/LManII, and MANBA/Beta-Man. Collectively, these enzymes participate in the breakdown of glycosaminoglycans (Fig 3). To further establish translational relevance, we next interrogated human cerebrospinal fluid proteomics from the Parkinson’s Progression Markers Initiative (PPMI) study. Of the 6 LSD gene modifiers with increased expression levels in αSyn flies, only MANBA was detected in the PPMI dataset. Interestingly, MANBA protein levels were significantly elevated in prodromal PD and subsequently reduced in clinically manifest PD (S9 Fig). Prodromal PD is defined as the presence of early disease biomarkers, but preceding clinical manifestations required for the diagnosis of PD (see Methods). Since gene loss-of-function in *Drosophila* enhances the αSyn locomotor phenotype, the increased protein expression of MANBA and other modifying genes may be consistent with a potential compensatory response to lysosomal stress (see below).

## Discussion

Mounting evidence supports an important connection between the genetic mechanisms of LSDs and PD. Using a cross-species, functional screening strategy, we discover 14 conserved human LSD genes with homologs that robustly enhance αSyn-mediated neurotoxicity when their activity is reduced in *Drosophila* models. The majority of these genes also show evidence for αSyn-independent requirements, and in several cases our data is suggestive of dose-sensitivity and context-dependent pleiotropy similar to *GBA* in PD and Gaucher disease. Two of the genes identified by our screen, *GBA* and *SMPD1*, are established PD risk genes [2,7,26], and our results confirm and extend data from other animal and cellular models (discussed below). In other cases, the evidence from human genetics may be more modest, but our discovery of genetic interactions with αSyn mechanisms increases the possibility that these genes may be *bona fide* PD risk factors. For example, in our prior analysis of exome sequencing data, *CTSD* and *SLC17A5* showed suggestive associations with PD risk [8]; however, the rarity of variants and available sample sizes have limited statistical power to confirm such loci. Our results also provide experimental support for LSD genes, such as *SCARB2* and *GNPTAB*, which are candidates at susceptibility loci from PD GWAS [10]. Indeed, GWAS rarely identify responsible genes definitively, but instead highlight regions that usually contain many viable candidates. As a group, we and others previously showed that genes causing LSDs harbor an aggregate burden of damaging rare variants among PD cases [8]. Here, using available GWAS data, we also demonstrate similar significant enrichment for more common variants associated with PD risk. Overall, the LSD genes prioritized by our functional screening strategy are outstanding candidates for further investigation as PD risk factors, including using both human genetics and experimental approaches.

Animal and cellular model experimental studies highlight several plausible mechanisms for how LSD genes might interact with and enhance αSyn-mediated neurotoxicity. First, LSD gene loss-of-function may impair turnover and thereby increase accumulation of toxic αSyn species. For example, *CTSD* encodes a lysosomal cathepsin, which can directly degrade αSyn [27,28]. More indirectly, reduced LSD gene activity may promote the accumulation of undigested substrates that secondarily accelerate αSyn misfolding or aggregation. In a variety of systems, glucosylceramide and its derivatives (e.g., glucosylsphingosine) have been shown to interact with αSyn in this manner [15,16]. Notably, based on a sensitive ELISA assay we did not detect any consistent evidence of elevated total αSyn protein levels following LSD gene knockdown, but it may be important to instead directly examine toxic, aggregated forms. Alternatively, it is possible that LSD gene dysfunction may also promote lysosomal stress and reduced autophagic flux [29,30]. However, we did not find support for this based on markers of lysosomal function, at least from studies conducted at a single, early timepoint.

In prior work, experimental manipulations of *GBA*, *SMPD1*, and *SCARB2—*which similarly cause sphingolipid storage disorders—have been demonstrated to induce accumulation and toxicity of αSyn [12,26,31]. Beyond sphingolipid/ceramide metabolism, our screen also identifies many fly homologs of genes causing human mucopolysaccharidoses (e.g., *GLB1*, *IDS*, *IDUA*, *MAN2B1*, *MANBA*). These disorders are characterized by accumulation of glycosaminoglycans, which are long chains of repeating, negatively charged disaccharide units that can be linked to protein cores to form proteoglycans. Though perhaps less well studied than sphingolipids, recent studies suggest that glycosaminoglycan metabolites have the potential to similarly influence αSyn aggregation [32]. In addition, proteoglycans are abundant at the cell surface and are a major constituent of the extracellular matrix, having recently been implicated in the propagation and/or internalization of pathologic αSyn species [33]. Interestingly, in one recent human genetic analysis, the genes causing mucopolysaccharidoses comprised a major driver for the rare variant burden association with PD risk among LSD genes [9]. Our screen also identifies homologs of two causes of cholesterol storage disorders, *LIPA* and *NPC1*. Cholesterol content may also be an important modulator of interactions between αSyn and the neuronal membrane, with consequences for synaptic transmission and PD pathologic progression [34,35]. In fact, conflicting epidemiologic studies have linked hypercholesterolemia to either increased [36] or decreased [37–39] PD risk, whereas other studies have found no such association [40]. To date, there has been no definitive genetic evidence for association of *NPC1* with PD risk [41,42]; however, subclinical metabolic perturbations [43,44] or parkinsonian signs [45,46] have been reported in heterozygous carriers of loss-of-function variants. Lastly, it is possible that disparate molecular pathways interact and feedback on one another within the lysosomal milieu. For example, loss of *NPC1* in Niemann Pick Disease type C leads to accumulation not only of cholesterol, but also secondary perturbations in sphingolipids and glycosaminoglycans [47]. Thus, it is conceivable that subtle perturbations affecting one enzymatic transformation might trigger a cascading metabolic failure that amplifies αSyn mediated neurotoxicity.

Ultimately, a successful mechanistic model must account for both the pleiotropic potential of LSD genes and their exquisite, dose-dependent interactions with αSyn-mediated PD mechanisms. Strikingly, whereas only a modest reduction of GCase enzymatic activity confers increased PD risk (e.g., ~75% residual function in carriers of the *GBA*^*E356K*^ allele) [48], Gaucher disease requires near complete loss of function (e.g., less than 15% residual GCase activity) [49]. Based on our screen, nearly all fly homologs of human LSD genes were capable of causing locomotor dysfunction independent of αSyn. This result is consistent with an obligate role for most LSD genes in the maintenance of CNS structure and function. By contrast, more modest loss-of-function in many LSD genes strongly enhanced the αSyn locomotor phenotype but caused little or no CNS dysfunction independent of αSyn. This was best exemplified by fly homologs of *NPC1* and *DNAJC5* (*Npc1a* and *Csp*, respectively) which both showed dominant, heterozygous enhancement of αSyn neurotoxicity, supporting dose-sensitive interactions. For many other genes, including the *Drosophila GBA* homolog, *Gba1b*, we also identified additional RNAi strains that support a synergistic interaction model. Prior studies using human αSyn transgenic flies have differed on potential synergistic interactions following *Gba1b* manipulation, but these studies have relied on either different alleles and tissue-specific drivers or distinct phenotypic assays [50,51]. Dose-sensitive interactions may arise from positive feedback between aging, αSyn toxicity, and progressive lysosomal dysfunction. Data from multiple experimental models highlight how αSyn can disrupt endosomal trafficking pathways, including delivery of GCase and other hydrolases to the lysosome [11,12], and thus may potentiate the impact of partial genetic loss-of-function in LSD genes. These pathologic interactions may be further amplified by aging, which among myriad cellular changes, is accompanied by reduced lysosomal proteostasis (autophagy) [52]. The impact of αSyn on the lysosome is suggested by our *Drosophila* proteomic profiles, revealing perturbations in the expression of numerous LSD proteins. In particular, the up-regulation detected for 6 modifier genes is consistent with a possible compensatory, albeit ineffective, cellular response, since genetic manipulation in the opposite direction (RNAi knockdown) worsened αSyn-induced locomotor impairment. Indeed, many lysosomal enzymes are transcriptionally coregulated, and prior studies support a well-conserved response pathway that can be activated in the context of lysosomal stress [53,54]. Notably, overexpression of either *Npc1a* or *Lip4* did not suppress the αSyn locomotor phenotype. Therefore, whereas knockdown of a given LSD gene in isolation appears sufficient to exacerbate neurodegeneration, targeted overexpression of a single enzyme may be ineffective to ameliorate a more widely distributed lysosomal defect.

The strengths of this study include the cross-species strategy, systematic consideration of multiple alleles for all LSD gene targets, longitudinal data collection, and an analytical framework that accounts for the potential impact of aging. The high-throughput locomotor screening assay is sensitive to early consequences of CNS dysfunction that precede cell death and subsequent structural degenerative changes. For *Npc1a* and *Lip4*, we additionally employed an independent assay for αSyn-induced retinal degeneration. One important potential limitation is that all genetic manipulations with RNAi were targeted exclusively to neurons. Many LSD genes, including *GBA*, are also expressed in glia, where they may also have important roles in the maintenance of CNS structure/function [55,56]. While we also tested classical mutant alleles, which are predicted to affect gene function globally, these reagents were only available for a subset of LSD gene homologs. The broad expression pattern for many LSD genes further complicates quantification of RNAi knockdown strength using neuron-specific drivers or comparison with heterozygous loss-of-function alleles. In the future it may also be important to directly assess neuronal cell death following interactions between LSD genes and αSyn toxicity, including cell-type specific degeneration of dopaminergic and other neurons. In addition, genes with negative results should be interpreted cautiously, since our interaction tests were potentially limited by the availability of RNAi reagents, which sometimes produce only a weak knockdown. Lastly, a minority of LSD genes are non-conserved in the *Drosophila* genome and were therefore not examined, including some with evidence for association with PD risk from human genetics (e.g., *ASAH1*, *GUSB*). Nevertheless, our results confirm and extend the strong genetic link between LSDs and PD and highlight many promising genes and metabolic pathways for further study in PD risk, pathogenesis, and therapy.

## Materials and methods

### LSD gene set enrichment analysis from PD GWAS

PD GWAS summary statistics [10] were analyzed using MAGMA v1.10 [17]. Gene location and European reference files for the GRCh37 genome build were downloaded from MAGMA webpage (https://ctg.cncr.nl/software/magma), and the BEDTools v2.26.0 [57] *intersect* function was used to interrogate SNPs from the PD GWAS summary statistics. MAGMA annotation, gene analysis and gene-set analysis steps were performed using default parameters. The list of LSD genes [8] (S1 Table) was used under the—*set-anot* parameter for the gene-set analysis, and selected genes were excluded for the sensitivity analysis. The X-linked LSD genes, *GLA*, *IDS*, and *LAMP2*, were excluded from GWAS, so summary statistics were not available for aggregate variants tests.

### Fly stocks and husbandry

Human *α-synuclein* transgenic lines with codon optimization for *Drosophila* were previously described [19]. For locomotor screening, a recombinant second chromosome line harboring 2 *UAS-α-synuclein* insertions was used, as in prior studies [21]. For retinal histology, a third chromosome insertion was employed, also from previous work [25]. The *GAL4-UAS* system [58] was used for ectopic expression of both αSyn and RNA-interference (RNAi) transgenes. For pan-neuronal expression, we used *elav*^*c155*^*-GAL4* [59], which is available from the Bloomington *Drosophila* Stock Center (BDSC; Bloomington, IN, USA). For expression in retinal photoreceptors, we used *Rh1-GAL4* (second chromosome insertion) [25,60]. For the locomotor screen of conserved LSD genes, virgin females obtained from RNAi or other allelic strains were crossed to male *elav*^*c155*^*-GAL4 / Y; UAS-SNCA / Cyo*,*GAL80*. To evaluate αSyn-independent effects, RNAi or other genetic strains, potential modifier strains were crossed with *elav*^*c155*^*-GAL4 / Y* or *w*^*1118*^ */ Y* males. Only female progeny carry both the *elav-GAL4* driver and the *UAS-SNCA* and/or the *UAS-RNAi* transgenes; therefore, female progeny were used for all experiments (e.g., *elav-GAL4 / +; UAS-SNCA / UAS-RNAi* or *elav-GAL4 / +; UAS-SNCA / allele*). All modifier gene manipulations (RNAi or alleles) were tested in heterozygosity. Crosses and progeny were established and maintained at 25°C. For secondary modifier tests using the αSyn retinal degeneration assay, all crosses were established at 18°C, and progeny were shifted to 25°C within 24 hours of eclosion and aged 15 days, following published protocols [25]. RNAi transgenic strains (*UAS-RNAi*) were obtained from the Vienna Drosophila Resource Center (VDRC; Vienna, Austria) [22], BDSC for the Harvard Transgenic RNAi Project [23], and the National Institute of Genetics Fly Stock Center (NIG; Japan). All RNAi lines used for this study are detailed in S3 Table. As a control, we used the VDRC *w*^*1118*^ genetic background strain (line 60000) and *UAS-RNAi-scramble* (*v2691*; VDRC gDNA plasmid dna451). Additional alleles for fly homologs of LSD genes, including transposon insertions, other classical mutation alleles, and overexpression transgenic lines, were obtained from BDSC or requested from other laboratories, including *crq*^*KO*^ [61], *dsb*^*KO*^ [62], *UAS-Lip4* [63], *UAS-Npc1a* [64], *Gba1b*^*KO*^ [65], and *Gba1a*,*b*^*KO*^ [65].

### Genetic screen

The *Drosophila* Integrated Ortholog Prediction Tool (DIOPT) [66] was used to identify all conserved fly homologs of human LSD genes. We required consensus from at least 3 independent bioinformatic algorithms for inclusion of a fly gene homolog (DIOPT score of 3 or greater). Where multiple paralogs met our criteria, we attempted to carry forward all such candidates. In the exceptional cases where more than 5 gene paralogs were identified as potential homologs for a given human gene, a more stringent DIOPT score cut-off was used (S1 Table). The screen was conducted in two stages. First, 174 RNAi strains from VDRC were obtained to knock down 86 unique homologs of human LSD genes (reagents were not available for 6 conserved genes). Locomotor behavior (see below) was assayed in female adult flies at up to 5 time points between 8 and 18 days post-eclosion. Based on the results, we subsequently obtained an additional 85 lines for further screening, including independent RNAi strains (from BDSC and NIG) and other available alleles. Lastly, for validation of screen results, all modifier genes supported by evidence from multiple independent allelic strains were re-evaluated in both the presence and absence of αSyn, and locomotor behavior was assayed at 11 or more time points over 30 days of aging.

### Robot-assisted locomotor assay

The negative geotaxis climbing assay was performed using a custom robotic system (SRI International, available in the Automated Behavioral Core at the Duncan Neurological Research Institute), as previously described [20]. Negative geotaxis is elicited by “tapping” vials of flies to knock them to the bottom of custom vials. After three taps, video cameras recorded and tracked the movement of animals at a rate of 30 frames per second for 7.5 seconds. For each genotype, 6–8 replicates of 15 female animals were tested in parallel (biological replicates), and each trial was repeated five times (technical replicates). Replicates were randomly assigned to positions throughout a 96-vial plate and were blinded to users throughout the duration of experiments. Quantification was based on the average climbing speed of flies included in each biological replicate. Speed of individual flies was computationally deconvoluted from video recordings. For configuration and running of the robotic assay and video acquisition, we used the following software packages: Adept desktop, Video Savant, MatLab with Image Processing Toolkit and Statistics Toolkit, RSLogix (Rockwell Automation), and Ultraware (Rockwell Automation). Additional custom software was developed for assay control (SRI graphical user interface for controlling the machine) and analysis [FastPhenoTrack (Vision Processing Software), TrackingServer (Data Management Software), ScoringServer (Behavior Scoring Software), and Trackviewer (Visual Tracking Viewing Software)].

### Adult retina histology

Fly heads from 15-day-old female animals were fixed in 8% glutaraldehyde and embedded in paraffin. Tangential retinal sections (3 μm) were cut using a Leica Microtome (RM2245) and stained with hematoxylin and eosin. Retinas from at least three animals were examined and quantified for each experimental genotype. Enhancement of αSyn-induced retinal degeneration was quantified based on the severity of retinal vacuolar changes seen in stained histologic sections. We examined representative photographs taken with a 40X objective from well-oriented, intact tangential sections at a depth in which the retina achieves maximal diameter. Using ImageJ software [67], we recorded the area occupied by all vacuoles with a diameter greater than 4μm and divided by the total retinal area to compute a percentage.

### RT-PCR

RNAi knockdown was examined for selected genes of interest, including those (1) related to cholesterol metabolism (*Npc1a*, *Lip4*); (2) synergistic enhancers with evidence from heterozygous loss-of-function alleles (*Npc1a*, *Csp*); and (3) genes with locomotor phenotypes consistent with possible dose-dependence responses to RNAi manipulations (*Gba1b*, *Dsb*, *Beta-Man*). 30 heads per replicate from 10-day-old female flies were homogenized in 1 mL TRIzol Reagent (Invitrogen # 15596026) and RNA was extracted using standard chloroform:phenol methods [68]. Reverse transcription was performed using SuperScript IV VILO Master Mix (Invitrogen # 11756050) using 2 μg RNA per reaction, according to the vendor protocol. cDNA solution was diluted 1:40 to obtain working concentration. Quantitative real-time PCR amplification were performed on 1 μL diluted cDNA in a 10 μL mixture containing SsoAdvanced Universal SYBR Green Supermix (BioRad # 1725271) and specific primers for each gene (see below) on the QuantStudio 5 Real-Time PCR System (Applied Biosystems #A34322). Amplification parameters were as follows: 2 minutes at 50°C, 10 min at 95°C, followed by 40 cycles of 15 sec at 95°C and 1 min at 60°C, with temperature adjustment rates of 1.6°C / second. Relative mRNA expression level was calculated by the threshold cycle (Ct) value of each PCR product and normalized to the average values of *GADPH* and *RPL32* housekeeping genes by using the comparative 2^−ΔΔCt^ method [69]. The following primer pairs were used:

RPL32 Forward:     ATCGGTTACGGATCGAACAA

RPL32 Reverse:     GACAATCTCCTTGCGCTTCT

GAPDH Forward:     TAAATTCGACTCGACTCACGGT

GAPDH Reverse:     CTCCACCACATACTCGGCTC

β-Man Forward:     GCGTTTTCCCATTGGCAACT

β-Man Reverse:     ACGTCGAACTGAAAGAAATTGGA

Csp Forward:     TGCGGCTGATAAGTTCAAGGA

Csp Reverse:     TTCTCCTCGCCAAACTGCTC

Dsb Forward:     AAAAACCAAATTGCAAGCAAGAADsb

Reverse:     CAAGCCCATAATTCAAGTGTTTCG

Gba1b Forward:     CACTGCTTGGCTTTCTTACTACA

Gba1b Reverse:     GCAGACACACACGCTTCCA

Lip4 Forwad:     CCTCAATTCCACGGGCGTAA

Lip4 Reverse:     TCAGCTTGAGGACCAACGAT

Npc1a Forward:     GCTGAAGAAACGCTGTGGATT

Npc1a Reverse:     GCACCAAGTTCTCCATGCAG

### Western blotting

Adult fly heads were homogenized in 2X Laemmli Sample Buffer (Bio-Rad) with 5% β-mercaptoethanol (Calbiochem) using a pestle (Argos Technologies). The lysates were heated at 95°C for 5 min, followed by centrifugation at 21,130 × *g* at 4°C for 15 min before SDS-PAGE analysis. Samples were loaded into 4–12% Bis-Tris gels (Invitrogen), separated by SDS-PAGE. Gels were transferred to PVDF membrane (Millipore), and blocked in Intercept (TBS) Blocking Buffer (LI-COR). We used the following primary antibodies and dilutions: mouse anti-tubulin (DM1A, Sigma Aldrich, RRID:AB_477583), 1∶1000; Mouse anti-CTSL (clone 193702, MAB22591, R and D Systems, RRID:AB_2087830), 1:2000; Rabbit anti-p62/Ref(2)p [70] 1:2000. IRDye secondary antibodies (LI-COR) were used in 1X TBST (Tris-buffered saline + 0.1% Tween-20) at 1:5000.

### Enzyme-linked immunosorbent assay (ELISA)

Homogenates were prepared from five 10-day-old female flies heads per replicate in 200 μL Denaturing Cell Extraction Buffer (Invitrogen # FNN0091) treated with 1 mM PMSF and protease inhibitor cocktail (Sigma-Aldrich Cat. No. P-2714) using a pestle. Samples were lysed for 30 minutes on ice and spun down at 13,000 rpm for 15 minutes at 4°C, per manufacturer instruction. To detect α-synuclein protein levels, the alpha Synuclein Human ELISA Kit (Invitrogen # KHB0061) was used according to manufacturer instruction. Briefly, homogenates were diluted 1:25 in ELISA Kit diluent buffer. 50 μL diluted homogenate and 50 μL Human Synuclein Detection Antibody were applied to each well of the provided antibody-coated microplate and incubated at room temperature for 3 hours. Plate was washed using provided 1X ELISA Wash Buffer, then incubated in 100 μL per well Anti-Rabbit IgG HRP for 30 minutes at room temperature. Plate was again washed and incubated in 100 μL Stabilized Chromogen per well for 30 minutes at room temperature in the dark. 100 μL Stop Solution was added to each well and signal intensity was measured at 450 nm on a FLUOstar OPTIMA plate reader (BMG LABTECH). Each ELISA was performed with 2 technical replicates per biological replicate, as recommended in the ELISA Technical Guide provided by Thermo Fisher Scientific. Standard curves were included for each plate using the provided purified α-synuclein standards.

### Cholesterol quantification

The Amplex Red Cholesterol Assay Kit (Invitrogen # A12216) was used for sterol detection in fly head homogenates. 5–10 fly heads per replicate from 10-day-old female flies were homogenized in 40 μL / head 1X Reaction Buffer from kit using a pestle. Homogenates were vortexed and incubated at 37°C for 10 minutes before spinning down at max rpm for 10 minutes at room temperature. Supernatant was gently mixed before aliquoting, to ensure incorporation of oily lipid layer. In a black polystyrene 96-well plate, 50 μL homogenate per well was combined with the assay kit working solution containing Amplex Red Reagent, HRP, and cholesterol oxidase in 1X Reaction Buffer, per manufacturer instructions. Plate was incubated protected from light for 60 minutes at 37°C. FLUOstar OPTIMA plate reader (BMG LABTECH) was used to measure fluorescence at excitation range of 530–560 nm and emission detection at 590 nm. 2 technical replicates were included for each biological replicate. Standard curves were included for each plate using the provided purified cholesterol standards.

### Proteomic analysis of LSD genes

For *Drosophila* proteomics, tandem mass tag mass-spectrometry proteomics was performed for αSyn transgenic and control flies, using the identical genotypes as the locomotor screen (*elav>aSyn* and *elav-GAL4 / +*) and following previously published protocols [71]. Homogenates were prepared from adult fly heads aged to 10 days, including 8 biological replicate samples per model consisting of approximately 50 heads each. The full dataset includes quantitation of 6,610 unique proteins and is included as supplemental data (S6 Table and S1 Dataset). Proteins containing missing values were excluded and protein intensity values mapping to the same flybase ID were summed. For this study, our analyses were restricted to 48 *Drosophila* proteins (S4 Table); a more comprehensive, proteome-wide analysis will be reported elsewhere. Differential protein expression was calculated with DESeq2 v1.34.0, as in prior work [72,73] using genotype as a linear regression covariate and using the *lfcShrink* function. The DESeq2 *counts* function was implemented in order to plot normalized abundance. For replication, we leveraged an independent proteomics dataset from *elav>aSyn* and *elav-GAL4 / +* flies, including n = 5 replicate samples and 6 aging timepoints between 2 and 21 days (60 samples total). The full dataset includes 6,128 detected proteins and is included as supplemental data (S7 Table and S2 Dataset).

For proteomic analyses from human CSF, we leveraged samples and data from the Parkinson’s Progression Markers Initiative (PPMI; www.ppmi-info.org). PPMI is a longitudinal observational study with comprehensive clinical and imaging data and biological samples. Blood, saliva, or DNA samples were used for genetic mutation testing of LRRK2 (G2019S and R1441G), GBA (N370S) and SNCA mutations. Proteomic data was generated on 1,075 samples using the aptamer-based SomaScan5K platform. A total of 4,783 analytes remain after QC: outliers were removed by 1.5 IQR threshold, aptamer and individual call rate <65%, and aptamer and individual call rate <85%. 917 samples were present with PPMI phenotypes including 185 control subjects without PD, 545 PD cases, and 187 subjects with prodromal PD. Prodromal PD is defined as the presence of REM sleep behavior disorder and/or genetic risk factors along with hyposmia and (in most cases) neuroimaging evidence of a dopamine transporter deficit, but these subjects lack clinical manifestations allowing the diagnosis of PD. MANBA had one analyte in the Soma5K dataset. 736 total PPMI samples had both diagnosis and protein data. Violin plots were generated in R via the *ggplot2* and *ggsignif* packages.

### Statistical analysis

MAGMA analysis was performed using default parameters. An F-test was performed to compute gene p-values. For gene set analyses, p-values are converted to Z-values, and a competitive analysis is performed, returning an overall T-test p-value. Data from our robotic *Drosophila* locomotor assay was processed by first calculating mean and standard deviation values for climbing speed across the replicates of each genotype tested using the *mean* and *sd* functions in R [74]. Each experimental genotype was compared against all control genotypes tested on the robotic assay at the same time for all downstream statistical analyses. Within-tray analyses were conducted to minimize differences between groups of flies (potential batch effects). We employed longitudinal mixed effects models in our analyses to better represent the age-dependent changes which we hypothesized were modified by differences in genotype. These models were implemented using the *lme4* package in R [75]. We used a random intercept term to model the mean climbing speed of each genotype and smoothing splines (cubic B-splines) to capture non-linear trends over time [76]. We tested the differences between all possible pairs of genotypes within each tray by testing the interactions between genotypes and their B-splines using one-way ANOVA (*aov* function in R) with three nested statistical models of increasing complexity: (i) genotype, (ii) genotype + time, and (iii) genotype*time. The genotype-only model examined mean shifts in climbing speed between genotypes without accounting for changes over time; the genotype+time model additionally considered non-linear time trends; and the genotype*time model also considered interactions leading to changes in spline slope between genotypes. We report p-values derived from all 3 models in S5 Table; plots show the p-value for the most complex statistical model (i, ii, or iii) meeting our significance threshold of α = 5x10^-5^.

We also applied longitudinal mixed effects models and the same statistical threshold (p<5x10^-5^) to identify those genetic manipulations (*elav>RNAi / +* or *allele / +*) causing a locomotor phenotype that was significantly different from *elav-GAL / +* control flies, independent of αSyn. Genetic manipulations that were not significantly different from controls (p>5x10^-5^) were classified as “no/mild” toxicity. For all others showing significant differences (p<5x10^-5^), we further classified the strength of phenotype by comparing the *elav>RNAi* (or *allele / +*) locomotor phenotype to that caused by *elav>αSyn*. Those genetic manipulations causing locomotor phenotypes that were less extreme than *elav>αSyn* were considered “moderate” toxicity. If the locomotor phenotype curve crossed the *elav>αSyn* curve to produce a more extreme climbing impairment, we considered this indicative of “severe” toxicity.

For the retinal histology assay, comparisons of vacuolar degenerative change were made using a 2-tailed student’s unpaired t-test followed by Dunnett’s post-hoc test for multiple comparisons, implemented in GraphPad Prism. For the αSyn ELISA, concentration of αSyn was estimated from fluorescence intensity using an interpolated standard curve. Comparisons of αSyn concentrations were made using ANOVA with Dunnett’s multiple comparisons post-hoc test, also in GraphPad Prism. Except as noted above, differential expression analysis of *Drosophila* proteomics data was performed using default DESeq2 parameters, which implements a Wald test, and differentially expressed proteins were filtered using a significance threshold of Benjamini-Hochberg adjusted p-value < 0.05. For visualization, t-tests were used to compare the normalized abundance levels between control and αSyn flies for each selected gene. For replication of *Drosophila* proteomics in the longitudinal dataset, we used linear regression with protein expression as an outcome, genotype as a predictor, and including age as a covariate: expression ~ genotype + age. Significance was computed using the Likelihood-ratio test, comparing to a base model: expression ~ age. Significance was set for a Benjamini-Hochberg adjusted p-value < 0.05. For the analysis of MANBA from human PPMI proteomics, the Wilcoxon rank sum test was implemented via the *geom_signif* function in the R package, *ggsignif*.

## Supporting information

S1 Fig Locomotor screen validation data. Data is shown for all modifiers of the α-synuclein (αSyn) locomotor phenotype. Pan-neuronal expression of human α-synuclein (Red: *elav > αSyn*) induces progressive locomotor impairment versus control flies (Green: *elav-GAL4* / +). Homologs were manipulated using RNA-interference (RNAi) or using loss-of-function alleles (allele). Each modifier gene was tested in heterozygosity, both in the presence (Purple: *elav>αSyn* + modifier) or absence (Blue: *elav>*RNAi or *elav-GAL4* + allele) of αSyn. Statistical comparisons based on one-way ANOVA considering three nested models (genotype, genotype + time, and genotype*time) and reporting results for the most complex model meeting significance. See also S5 Table for detailed statistical output. Significance testing examined whether modifier genes enhance the αSyn-induced locomotor impairment [p(+ syn)] and whether gene manipulations cause locomotor phenotypes independent of αSyn [p(- syn)]. The 2 comparisons (*i*. and *ii*.) are indicated on the second plot shown (A, *Npc1a*^*1*^). We classified modifier strains based on the severity of phenotype produced independent of αSyn. Genetic manipulations that were not significantly different from controls (p>5x10^-5^) were classified as “no/mild” toxicity (A). For all others showing significant differences (p<5x10^-5^), we further classified the strength of phenotype by comparing the *elav>RNAi* (or *allele / +*) locomotor phenotype to that caused by *elav>αSyn*. Those genetic manipulations causing locomotor phenotypes that were less extreme than *elav>αSyn* were considered “moderate” (B). If the locomotor phenotype curve crossed the *elav>αSyn* curve to produce a more extreme climbing impairment, we considered this indicative of “severe” toxicity (C).
(PDF)

S2 Fig Validation of RNA-interference (RNAi) strains and other alleles. Reverse transcription polymerase chain reaction (RT-PCR) was performed to confirm RNAi knockdown of selected genes. Total mRNA was prepared from the heads of 10-day old female flies. RNAi transgenes or alleles were tested in heterozygosity (e.g., *Elav-GAL4 / +; UAS-RNAi* or *allele / +*). The following RNAi strains were tested: *Gba1b* (KD1: *v21336*, KD2: *v101212*); *ß-Man* (KD1: *12582R-2*, KD2: *v110464*); *Csp* (KD1: *6395R-2*, KD2: *v103201*); *Dsb* (KD1: *v4100*, KD2: *v100219*); *Lip4* (KD1: *v31021*, KD2: *v106614*); *Npc1a* (*v105405*). Quantification based on analysis of at least n = 4 animals per genotype. Statistical comparisons were made using unpaired t-tests, followed by Dunnett’s post-hoc test. Error bars represent the standard error of the mean. **, p<0.01; ***, p<0.001; ****, p<0.0001; ns, non-significant
(TIF)

S3 Fig Overexpression of Lip4 and Npc1a. Pan-neuronal overexpression of *Lip4* (A) or *Npc1a* (B) using the *elav-GAL4* driver mildly enhances the α-synuclein locomotor phenotype. *Npc1a* overexpression caused locomotor impairment independent of α-synuclein. Climbing speed was assessed longitudinally, including at least 11 aged time points over 30 days (n > 6 replicates of 15 animals each). As in S1 Fig, statistical comparisons were based on one-way ANOVA, examining whether genetic manipulations modify locomotor behavior either in the presence [p(+ syn)] or absence [p(- syn)] of α-synuclein.
(TIF)

S4 Fig Analysis of cholesterol levels following manipulation of storage disorder genes. Cholesterol and cholesterol esters are modestly increased following *Npc1a* (left) or *Lip4* (right) loss-of-function. Homogenates were prepared from heads of 10-day old female flies. All RNAi transgenes or alleles were tested in heterozygosity and in the presence of the *elav-GAL4* pan-neuronal driver. The following RNAi strains were tested: *Npc1a* (*v105405*), *Lip4* (KD1: *v31021*; KD2: *v106614*). Quantification based on analysis of at least n = 5 replicate samples per genotype. Statistical comparisons were made using unpaired t-tests, followed by Dunnett’s post-hoc test. Error bars represent the standard error of the mean. *, p<0.05; **, p<0.01; ****, p<0.0001
(TIF)

S5 Fig Analysis of lysosomal function following manipulation of lysosomal storage disorder genes. Markers of lysosomal function, including autophagic flux (A, p62) or Cathepsin L (CTSL) proteolysis (B, C), was assayed following *Npc1a* or *Lip4* loss-of function. Gene knockdown using RNA interference (RNAi) transgenes or loss-of-function alleles were tested in heterozygosity with *elav-GAL4* and in either the presence or absence of α-synuclein. Western blots were performed on homogenates prepared from heads of 10-day old female flies and probed for p62 (A) or CTSL (B, C). The native CTSL proform, proCTSL (B), is cleaved in the acidic environment of the lysosome to generate the mature form, mCTSL (C). *Npc1a*^*1*^ caused an increase in both proCTSL and mCTSL, but this result was not seen consistently for other alleles or RNAi. The following RNAi strains were tested: *Npc1a* (*v105405*), *Lip4* (KD1: *v31021*; KD2: *v106614*). We used the *v2691* strain as a non-targeting, scramble RNAi. Quantification based on analysis of at least n = 4 replicate samples per genotype. Statistical comparisons were made using unpaired t-tests, followed by Dunnett’s post-hoc test. Error bars represent the standard error of the mean. ***, p<0.001; ****, p<0.0001 See S6 Fig for original western blot data.
(TIF)

S6 Fig Markers of lysosomal function following manipulation of lysosomal storage disorder genes. Original western blot data is shown for investigations of markers of lysosomal function, including autophagic flux (p62) or Cathepsin L (CTSL) proteolysis, following *Npc1a* or *Lip4* loss-of function. The following RNAi strains were tested: *Npc1a* (*v105405*), *Lip4* (KD1: *v31021*; KD2: *v106614*). We used the *v2691* strain as a non-targeting, scramble RNAi. See S5 Fig for quantitation and statistical analysis of these data. For quantitation, all intensity data was normalized to mean intensity of *elav>syn* bands on each blot, permitting an integrated analysis. Poorly-transferred p62 bands were excluded from analysis, including all of the *elav/+* control lanes on the *Npc1a*^*57*^ blot (middle row, right) and one of the *elav>Lip4*^*KD2/+*^ control lanes (top row, right).
(TIF)

S7 Fig Studies of α-synuclein protein expression. Enzyme linked immunosorbent assays (ELISA) were performed for quantification of total α-synuclein levels from 10-day-old fly head homogenates. α-synuclein was expressed pan-neuronally using the *elav-GAL4* driver (*elav>* α*syn*). LSD gene modifiers were manipulated using RNA interference knockdown (RNAi) or loss-of-function alleles; all such manipulations were tested in heterozygosity. (A) α-synuclein protein is sensitively and specifically detected by the ELISA in *elav>* α*syn* flies but not wildtype controls. α-synuclein protein levels are unchanged following co-expression of a non-targeting *UAS-RNAi*-*scramble* control construct. (B) Manipulations of either *Npc1a*, *Lip4*, or *Csp* do not cause any consistent changes in α-synuclein protein levels. The following RNAi strains were tested: *Npc1a* (*v105405*), *Lip4* (KD1: *v31021*; KD2: *v106614*), *Csp* (KD1: *6395R-2*, KD2: *v103201*). (C) For all other LSD gene modifiers, the RNAi transgene generating the strongest locomotor phenotype was selected for ELISAs: *Ect3* (*3132R-2*); *Idua* (*v13244*); *Dsb* (*v4100*); *ß-Man* (*v15028*); *Snmp1* (*v42496*); *Gba1b* (*v101212*); *CG15533* (*v102842*); *Ids* (*v105970*); *LManII* (*v108218*); *CG10104* (*v108431*); *Gntap* (*v109400*); *MFS3* (*v330237*). Only knockdown of *LManII* caused a significant increase in α-synuclein protein levels. Reduction of *MFS3* decreased α-synuclein protein levels. All other manipulations did not significantly impact α-synuclein protein levels. Quantification based on analysis of at least n = 5 replicate samples per genotype. Statistical comparisons were made using unpaired t-tests, followed by Dunnett’s post-hoc test. Error bars represent the standard error of the mean. *, p<0.05; **, p<0.01; ****, p<0.0001
(TIF)

S8 Fig Additional studies of α-synuclein-induced retinal degeneration. Representative images of retinal histology sections (A) and quantification (B) of additional *Npc1a* alleles and *Lip4* RNAi (*v106614*), showing consistent enhancement of α-synuclein-mediated neurodegeneration. Quantification based on extent of vacuolar changes (vacuole area / total area) from at least n = 3 animals per genotype. Statistical comparisons were made using unpaired t-tests, followed by Dunnett’s post-hoc test. Error bars represent the standard error of the mean. **, p<0.01; ***, p<0.001; ns, non-significant; Scale bar = 20μm.
(TIF)

S9 Fig Replication of lysosomal storage disorder protein differential expression. (A, B) Adult fly head homogenates were prepared from *elav>αSyn* or controls (*elav-GAL4 / +*), and Tandem Mass Tag proteomics were performed. (A) Cross-sectional comparisons of mean abundance from 10-day-old adults were analyzed using t-tests, considering n = 5 replicate samples for each genotype. Error bars represent the standard error of the mean. *, p<0.05; **, p<0.01; ***, p<0.001. (B) Complementary longitudinal analysis, including 6 aging timepoints between 2 and 21 days. Regression models considered protein expression as an outcome, genotype as a predictor, and included age as a covariate: expression ~ genotype + age. Significance was computed using the Likelihood-ratio test, comparing to the base model: expression ~ age. Benjamini-Hochberg adjusted p-value is shown. log2FC, log base2 (fold-change). (C) MANBA protein levels were examined from human cerebrospinal fluid from the Parkinson’s Progression Markers Initiative (PPMI), including 365 PD cases, 177 controls, and 111 prodromal PD cases, in which early disease biomarkers are present, but clinical manifestations are lacking for the diagnosis. MANBA was significantly elevated in prodromal PD and reduced in clinically manifest PD.
(TIF)

S1 Table Common variants associated with Parkinson’s disease (PD) risk are enriched in lysosomal storage disorder (LSD) genes. The multi-marker analysis of genomic annotation (MAGMA) tool [17] was used to test for enrichment among LSD gene sets (top) or 51 separate genes (bottom). As a sensitivity analysis, the LSD gene set tests were repeated following exclusion of either *GBA* or the top 3 genes (*GBA*, *SCARB2*, *IDUA*).
(XLSX)

S2 Table Conservation of Lysosomal Storage Disorder Genes in Drosophila. Human and fly gene homologs are shown, along with level of conservation based on the Drosophila Integrated Ortholog Prediction Tool [66]. 86 fly gene homologs were tested in this study. For 6 genes, genetic reagents were not available for testing: *Fuca*, *Hexo2*, *CG42638*, *Ppt1*, *CG42638* and *CG40006*.
(XLSX)

S3 Table Genetic reagents for manipulation of fly homologs of human lysosomal storage disorder genes. 259 genetic strains targeting 86 fly genes were tested, including RNA-interference (RNAi) and classical loss-of-function alleles. Detailed genotype information, the source of each line, and FlyBase ID are noted. Note: some of the RNAi lines tested in this study have been discarded by stock centers and may no longer be available. NA, not applicable.
(XLSX)

S4 Table Fly homologs of proteins encoded by human lysosomal storage disorder (LSD) genes are dysregulated in α-synuclein (αSyn) transgenic flies. Results from differential expression analysis are shown, based on mass spectrometry proteomics from αSyn vs. control flies. 15 out of 22 differentially-expressed proteins were up-regulated in αSyn flies.
(XLSX)

S5 Table Detailed statistical output from locomotor testing. (XLSX)

S6 Table Drosophila Mass-spectrometry Proteomics Discovery Study Metadata. Sample identifiers are noted for control (Ctrl: *elav-GAL4 / +*) and α-synuclein transgenic (Exp) flies. 8 replicates for each genotype were included in this mass-spectrometry proteomics study with tandem mass tags. For full dataset see S1 Dataset.
(XLSX)

S7 Table Drosophila Mass-spectrometry Proteomics Replication Study Metadata. Sample identifiers and ages (days) are noted for control (elav-GAL4 / +) and α-synuclein transgenic flies. 5 replicates for each genotype were included in this longitudinal study. For full dataset see S2 Dataset.
(XLSX)

S1 Dataset Drosophila Proteomics Discovery Study. (TXT)

S2 Dataset Drosophila Proteomics Replication Study. (CSV)

S3 Dataset Primary data for graphical and statistical analyses. (XLSX)
