# Supplementary material for: Functional screening of lysosomal storage disorder genes identifies modifiers of alpha-synuclein neurotoxicity
Source: PLoS Genet. 2023 May 18;19(5):e1010760. doi: 10.1371/journal.pgen.1010760 (PMC10231792; doi:10.1371/journal.pgen.1010760)
Supplement: S1 Fig — Data is shown for all modifiers of the α-synuclein (αSyn) locomotor phenotype. Pan-neuronal expression of human α-synuclein (Red: elav > αSyn) induces progressive locomotor impairment versus control flies (Green: elav-GAL4 / +). Homologs were manipulated using RNA-interference (RNAi) or using loss-of-function alleles (allele). Each modifier gene was tested in heterozygosity, both in the presence (Purple: elav>αSyn + modifier) or absence (Blue: elav>RNAi or elav-GAL4 + allele) of αSyn. Statistical comparisons based on one-way ANOVA considering three nested models (genotype, genotype + time, and genotype*time) and reporting results for the most complex model meeting significance. See also S5 Table for detailed statistical output. Significance testing examined whether modifier genes enhance the αSyn-induced locomotor impairment [p(+ syn)] and whether gene manipulations cause locomotor phenotypes independent of αSyn [p(- syn)]. The 2 comparisons (i. and ii.) are indicated on the second plot shown (A, Npc1a1). We classified modifier strains based on the severity of phenotype produced independent of αSyn. Genetic manipulations that were not significantly different from controls (p>5x10^-5^) were classified as “no/mild” toxicity (A). For all others showing significant differences (p<5x10^-5^), we further classified the strength of phenotype by comparing the elav>RNAi (or allele / +) locomotor phenotype to that caused by elav>αSyn. Those genetic manipulations causing locomotor phenotypes that were less extreme than elav>αSyn were considered “moderate” (B). If the locomotor phenotype curve crossed the elav>αSyn curve to produce a more extreme climbing impairment, we considered this indicative of “severe” toxicity (C). (PDF) [file pgen.1010760.s001.pdf]

(A) no / mild toxicity independent of αSyn

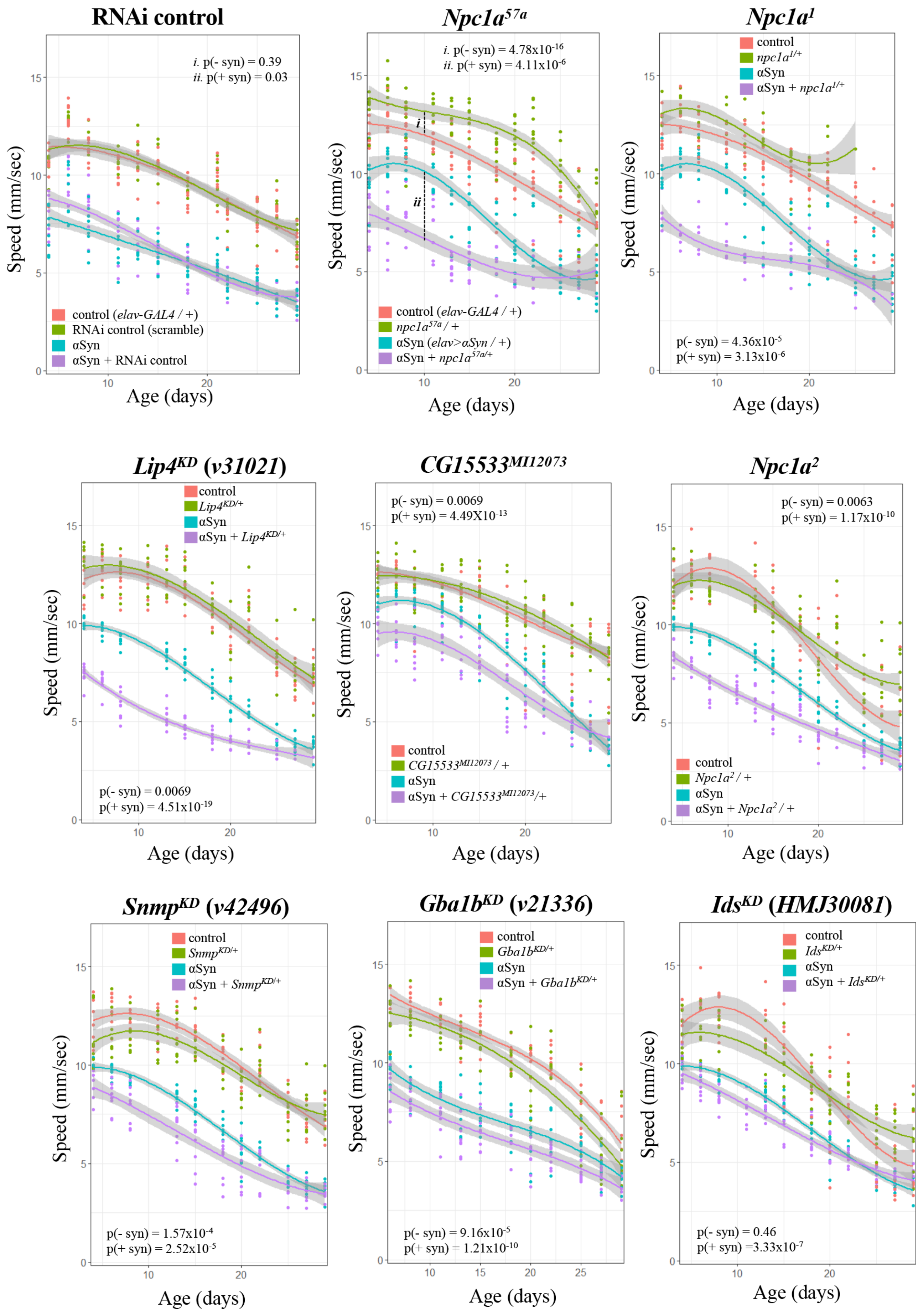

(B) moderate toxicity independent of αSyn

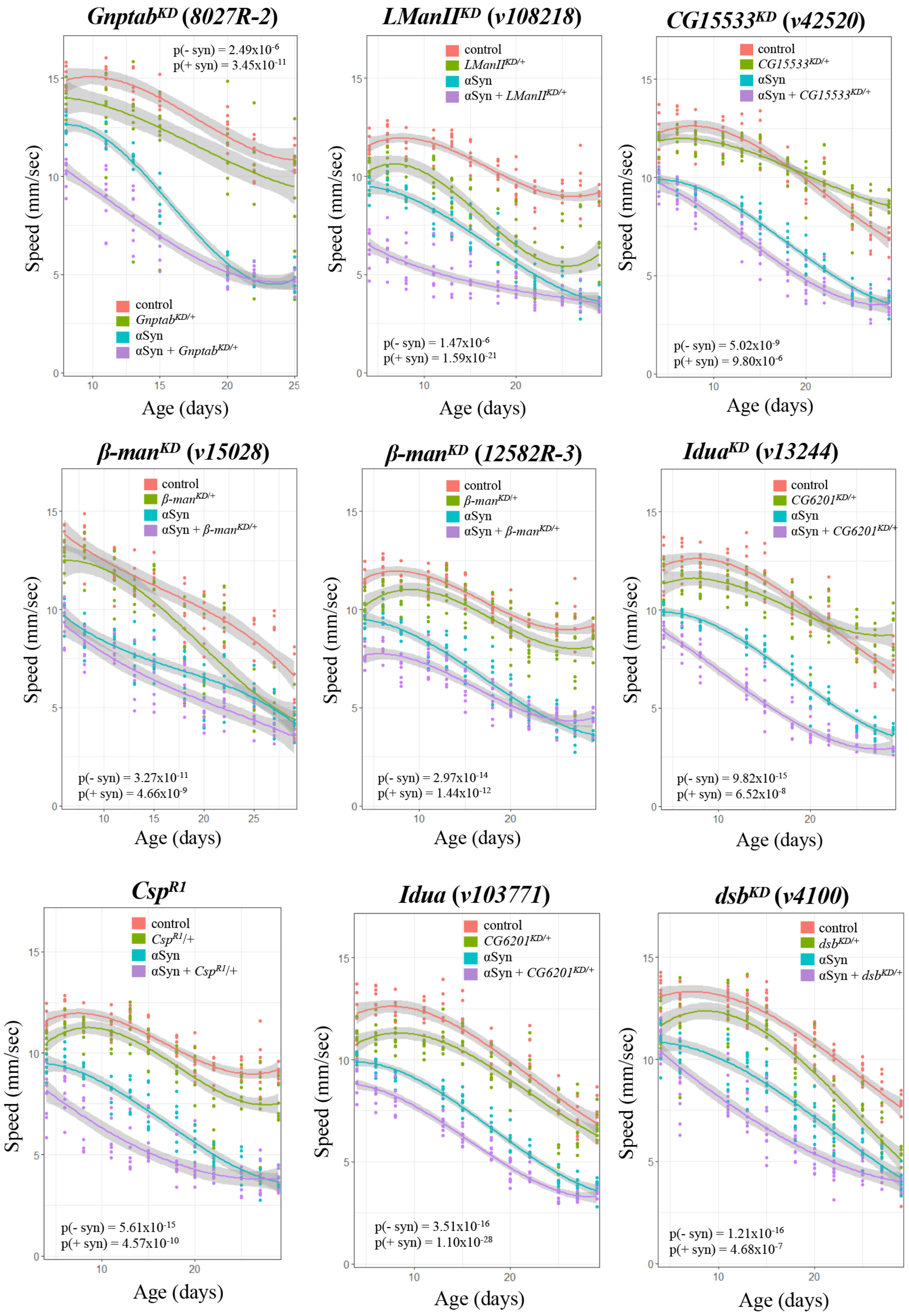

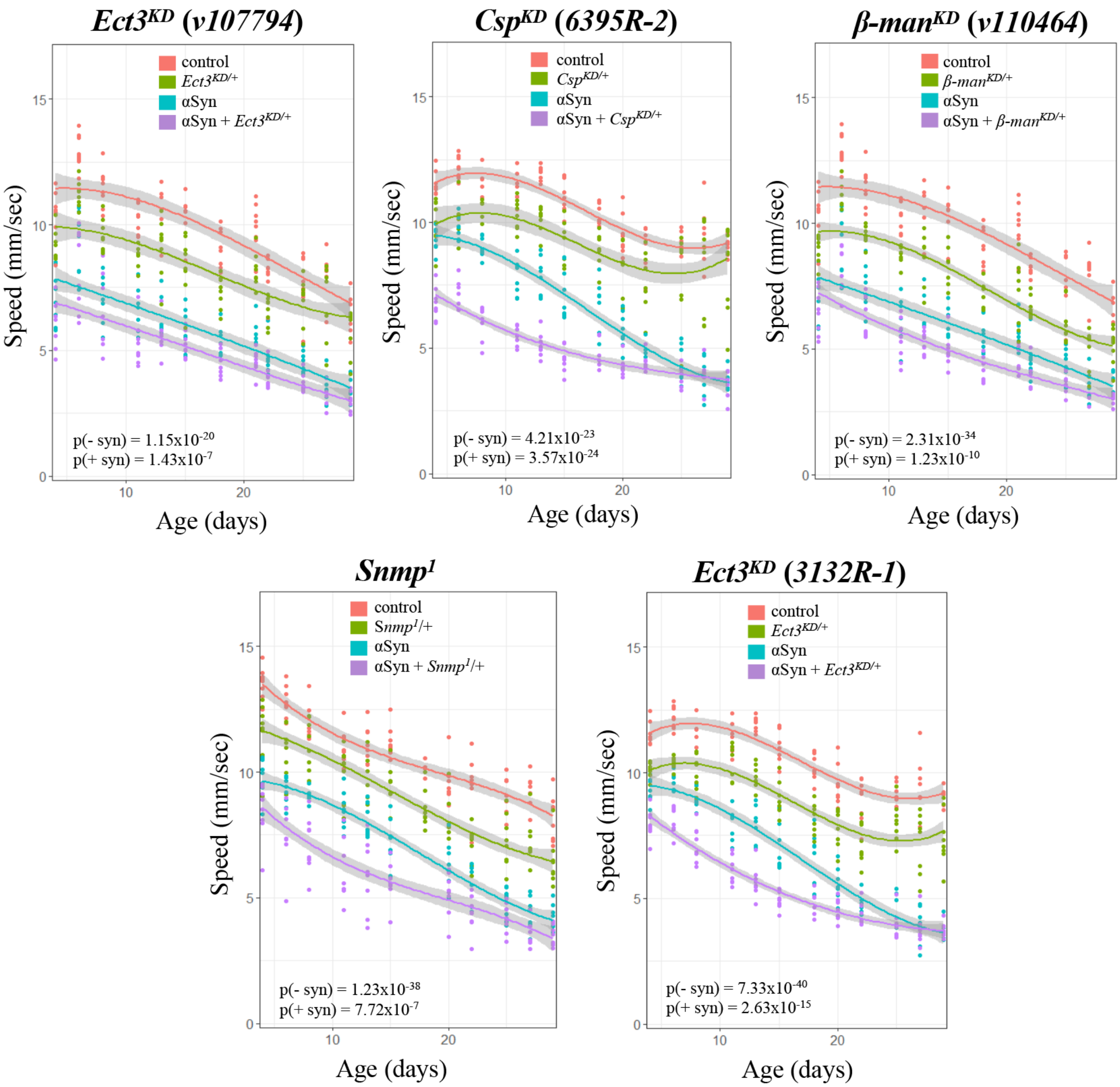

Ect3^{KD} (*v107794*)
control
Ect3$^{KD/+}$
αSyn
αSyn + *Ect3$^{KD/+}$*
Speed (mm/sec)
Age (days)
p(- syn) = 1.15x10^{-20}
p(+ syn) = 1.43x10^{-7}
Csp^{KD} (*6395R-2*)
control
Csp$^{KD/+}$
αSyn
αSyn + *Csp$^{KD/+}$*
Speed (mm/sec)
Age (days)
p(- syn) = 4.21x10^{-23}
p(+ syn) = 3.57x10^{-24}
β-man^{KD} (*v110464*)
control
β-man$^{KD/+}$
αSyn
αSyn + *β-man$^{KD/+}$*
Speed (mm/sec)
Age (days)
p(- syn) = 2.31x10^{-34}
p(+ syn) = 1.23x10^{-10}
Snmp^{1}
control
S*nmp^{1}*/+
αSyn
αSyn + *Snmp^{1}*/+
Speed (mm/sec)
Age (days)
p(- syn) = 1.23x10^{-38}
p(+ syn) = 7.72x10^{-7}
Ect3^{KD} (*3132R-1*)
control
Ect3$^{KD/+}$
αSyn
αSyn + *Ect3$^{KD/+}$*
Speed (mm/sec)
Age (days)
p(- syn) = 7.33x10^{-40}
p(+ syn) = 2.63x10^{-15}

(C) severe toxicity independent of αSyn

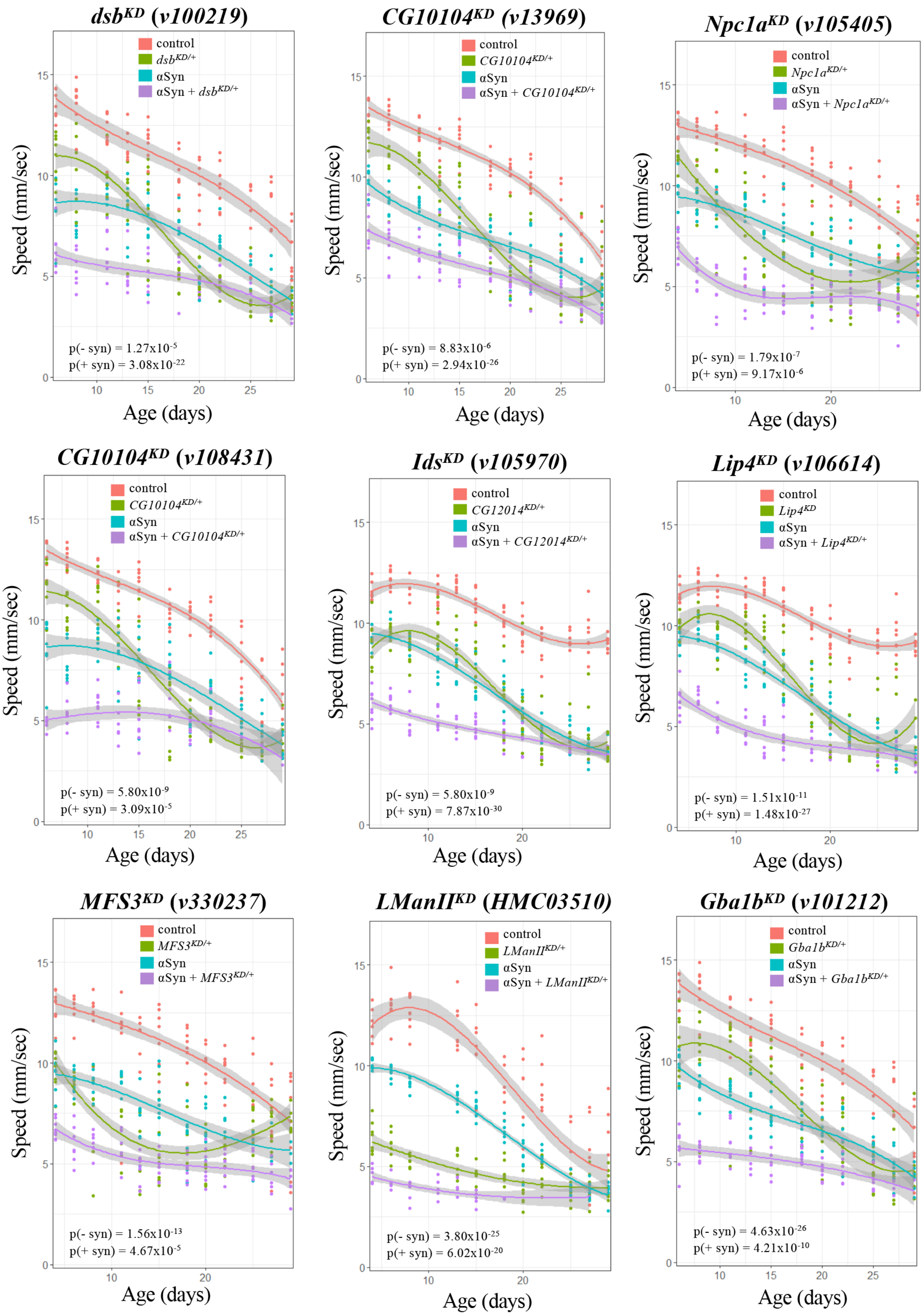

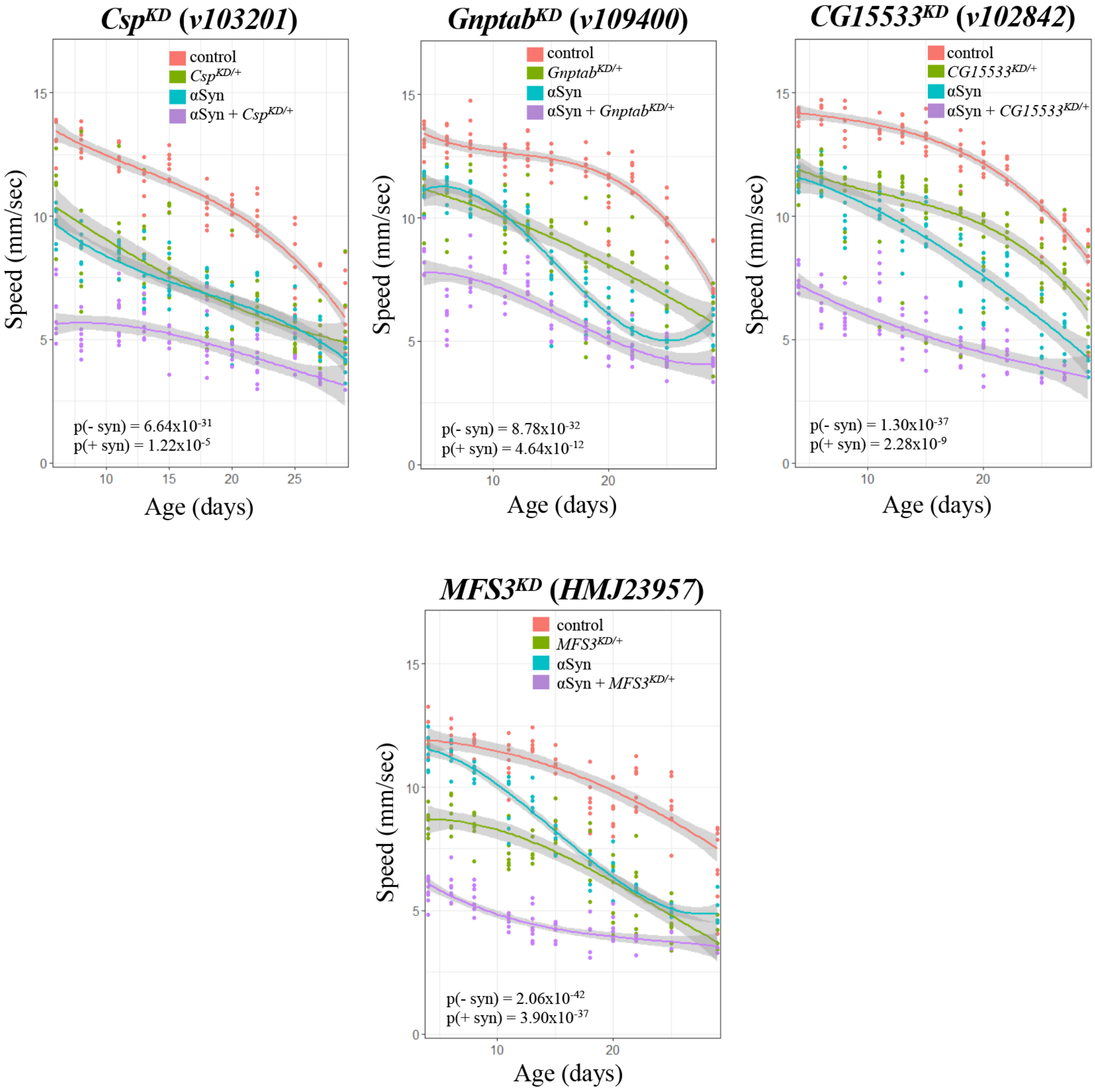

CspKD (v103201)
control
CspKD/+
αSyn
αSyn + CspKD/+
Speed (mm/sec)
Age (days)
p(- syn) = 6.64x10-31
p(+ syn) = 1.22x10-5
GnptabKD (v109400)
control
GnptabKD/+
αSyn
αSyn + GnptabKD/+
Speed (mm/sec)
Age (days)
p(- syn) = 8.78x10-32
p(+ syn) = 4.64x10-12
CG15533KD (v102842)
control
CG15533KD/+
αSyn
αSyn + CG15533KD/+
Speed (mm/sec)
Age (days)
p(- syn) = 1.30x10-37
p(+ syn) = 2.28x10-9
MFS3KD (HMJ23957)
control
MFS3KD/+
αSyn
αSyn + MFS3KD/+
Speed (mm/sec)
Age (days)
p(- syn) = 2.06x10-42
p(+ syn) = 3.90x10-37
