# Supplementary figures and images for: Functional screening of lysosomal storage disorder genes identifies modifiers of alpha-synuclein neurotoxicity
Source: PLoS Genet. 2023 May 18;19(5):e1010760. doi: 10.1371/journal.pgen.1010760 (PMC10231792; doi:10.1371/journal.pgen.1010760)

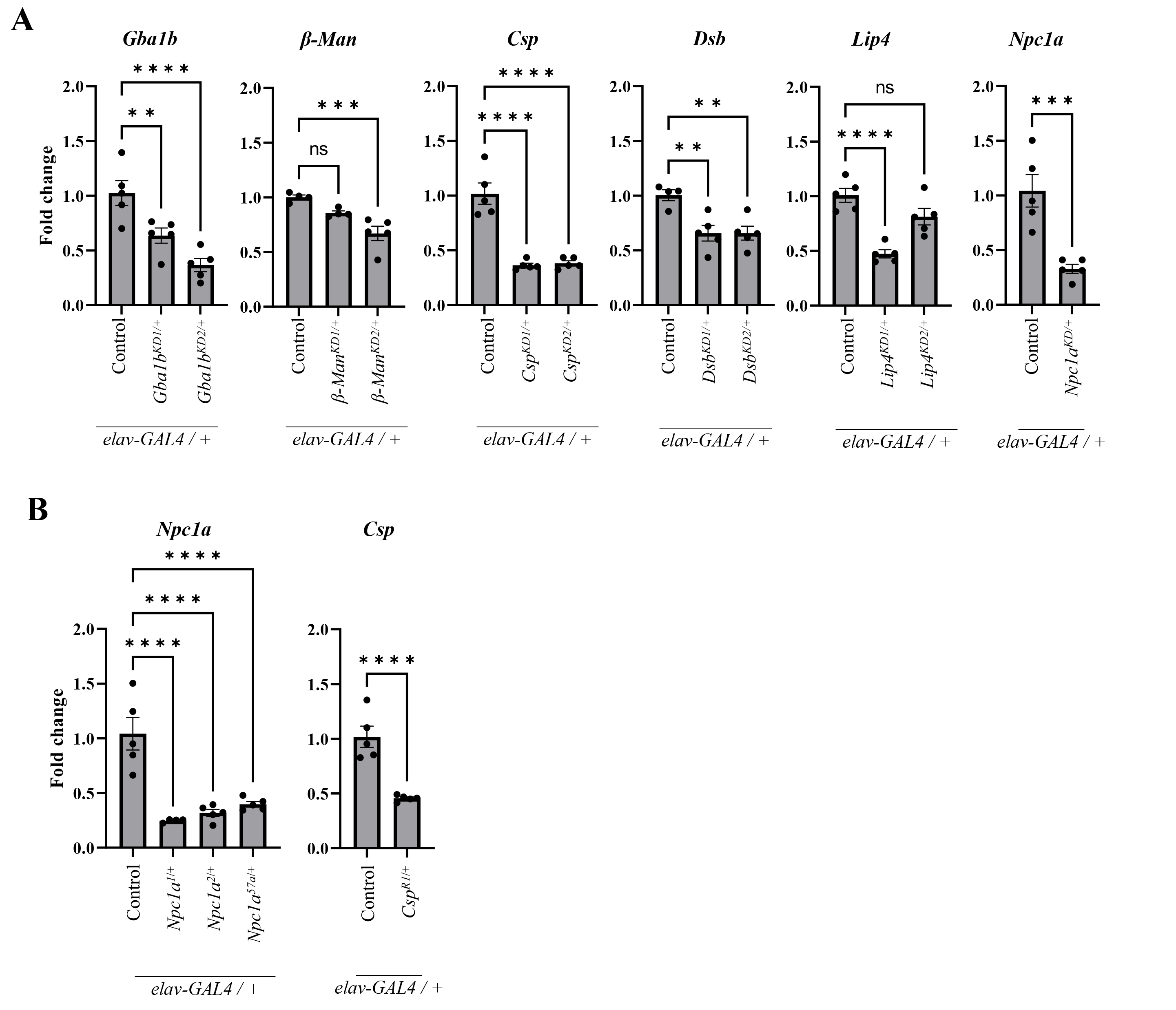

Supplement: S2 Fig — Reverse transcription polymerase chain reaction (RT-PCR) was performed to confirm RNAi knockdown of selected genes. Total mRNA was prepared from the heads of 10-day old female flies. RNAi transgenes or alleles were tested in heterozygosity (e.g., Elav-GAL4 / +; UAS-RNAi or allele / +). The following RNAi strains were tested: Gba1b (KD1: v21336, KD2: v101212); ß-Man (KD1: 12582R-2, KD2: v110464); Csp (KD1: 6395R-2, KD2: v103201); Dsb (KD1: v4100, KD2: v100219); Lip4 (KD1: v31021, KD2: v106614); Npc1a (v105405). Quantification based on analysis of at least n = 4 animals per genotype. Statistical comparisons were made using unpaired t-tests, followed by Dunnett’s post-hoc test. Error bars represent the standard error of the mean. **, p<0.01; ***, p<0.001; ****, p<0.0001; ns, non-significant (TIF) [file pgen.1010760.s002.tif]

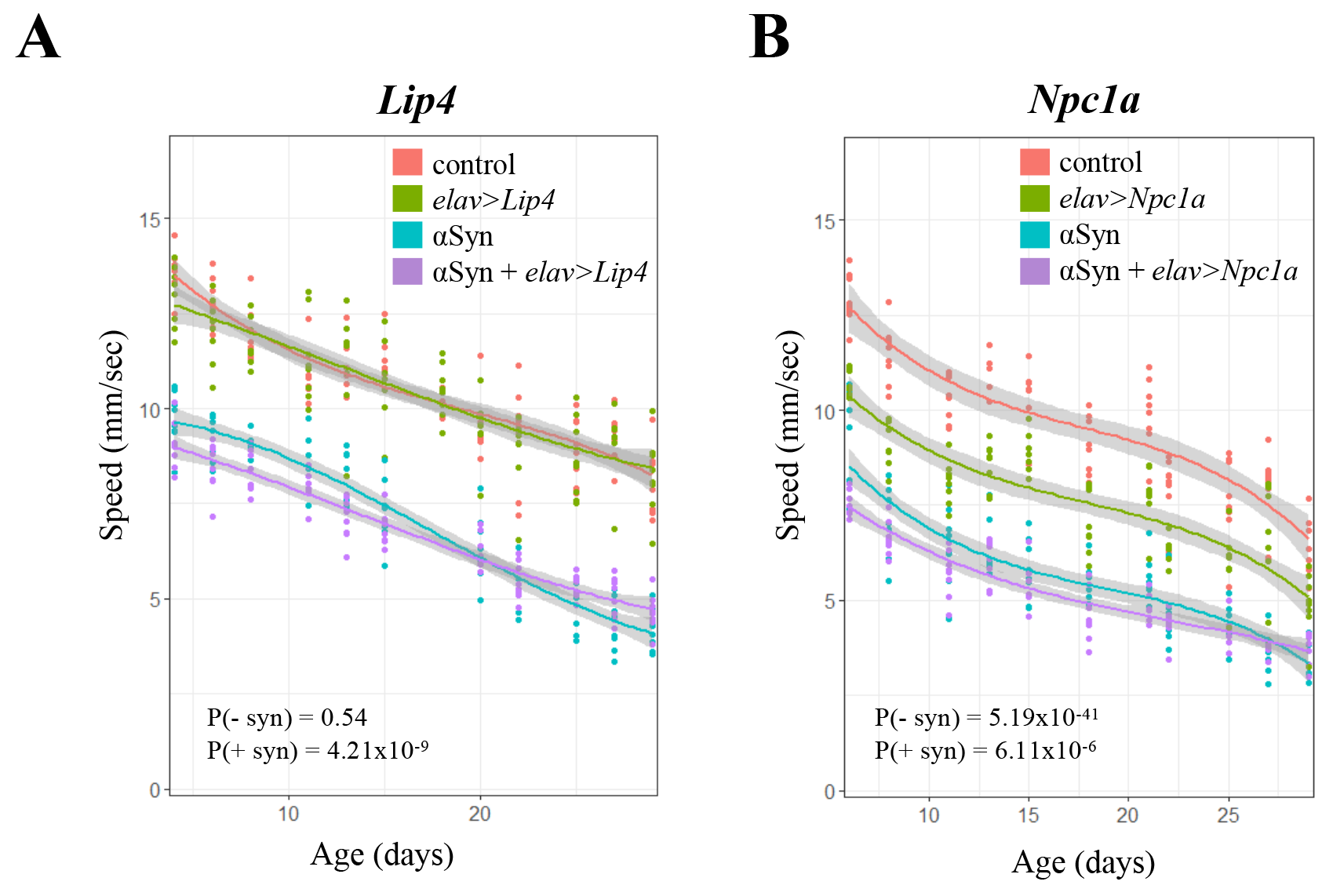

Supplement: S3 Fig — Pan-neuronal overexpression of Lip4 (A) or Npc1a (B) using the elav-GAL4 driver mildly enhances the α-synuclein locomotor phenotype. Npc1a overexpression caused locomotor impairment independent of α-synuclein. Climbing speed was assessed longitudinally, including at least 11 aged time points over 30 days (n > 6 replicates of 15 animals each). As in S1 Fig, statistical comparisons were based on one-way ANOVA, examining whether genetic manipulations modify locomotor behavior either in the presence [p(+ syn)] or absence [p(- syn)] of α-synuclein. (TIF) [file pgen.1010760.s003.tif]

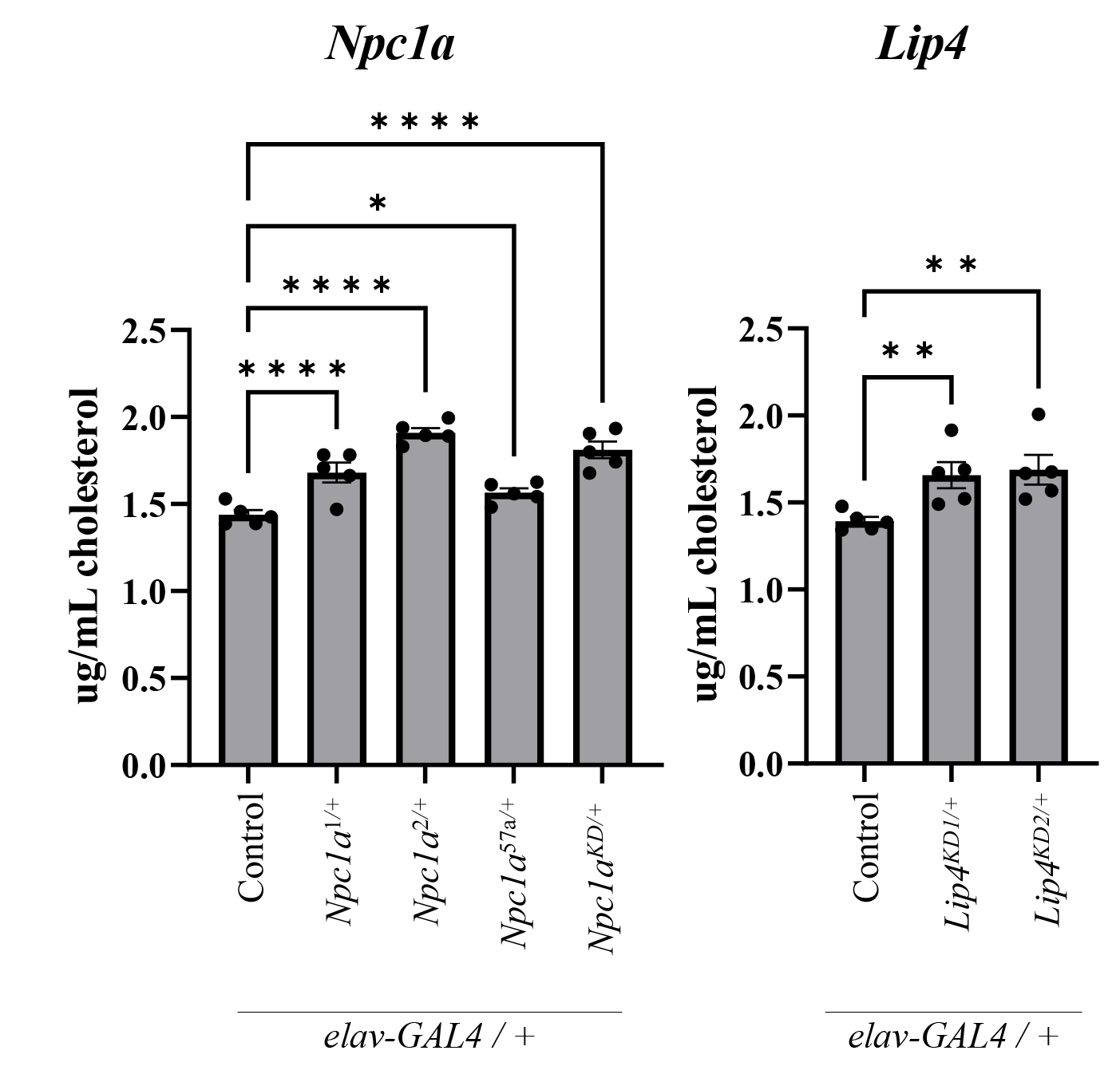

Supplement: S4 Fig — Cholesterol and cholesterol esters are modestly increased following Npc1a (left) or Lip4 (right) loss-of-function. Homogenates were prepared from heads of 10-day old female flies. All RNAi transgenes or alleles were tested in heterozygosity and in the presence of the elav-GAL4 pan-neuronal driver. The following RNAi strains were tested: Npc1a (v105405), Lip4 (KD1: v31021; KD2: v106614). Quantification based on analysis of at least n = 5 replicate samples per genotype. Statistical comparisons were made using unpaired t-tests, followed by Dunnett’s post-hoc test. Error bars represent the standard error of the mean. *, p<0.05; **, p<0.01; ****, p<0.0001 (TIF) [file pgen.1010760.s004.tif]

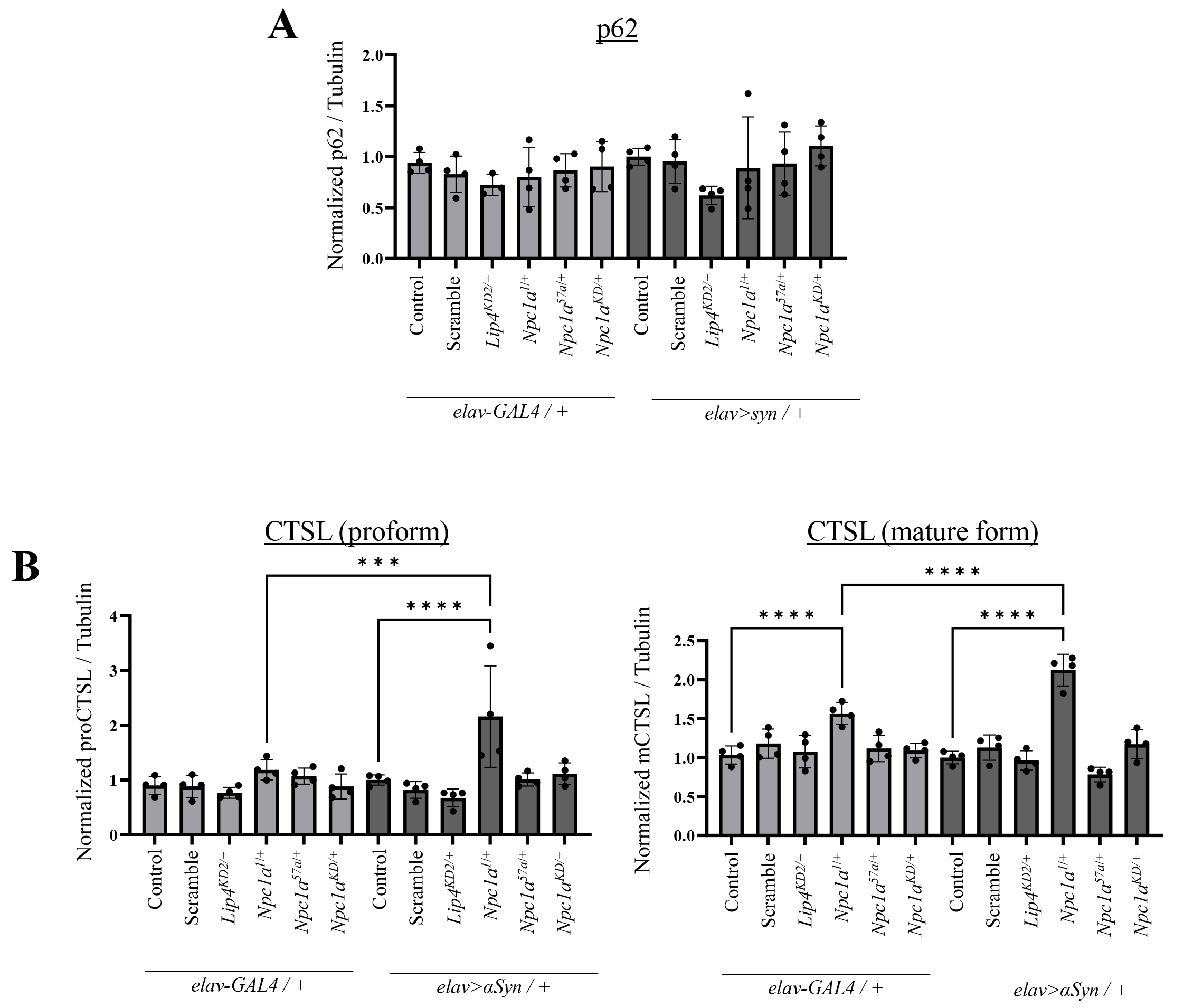

Supplement: S5 Fig — Markers of lysosomal function, including autophagic flux (A, p62) or Cathepsin L (CTSL) proteolysis (B, C), was assayed following Npc1a or Lip4 loss-of function. Gene knockdown using RNA interference (RNAi) transgenes or loss-of-function alleles were tested in heterozygosity with elav-GAL4 and in either the presence or absence of α-synuclein. Western blots were performed on homogenates prepared from heads of 10-day old female flies and probed for p62 (A) or CTSL (B, C). The native CTSL proform, proCTSL (B), is cleaved in the acidic environment of the lysosome to generate the mature form, mCTSL (C). Npc1a1 caused an increase in both proCTSL and mCTSL, but this result was not seen consistently for other alleles or RNAi. The following RNAi strains were tested: Npc1a (v105405), Lip4 (KD1: v31021; KD2: v106614). We used the v2691 strain as a non-targeting, scramble RNAi. Quantification based on analysis of at least n = 4 replicate samples per genotype. Statistical comparisons were made using unpaired t-tests, followed by Dunnett’s post-hoc test. Error bars represent the standard error of the mean. ***, p<0.001; ****, p<0.0001 See S6 Fig for original western blot data. (TIF) [file pgen.1010760.s005.tif]

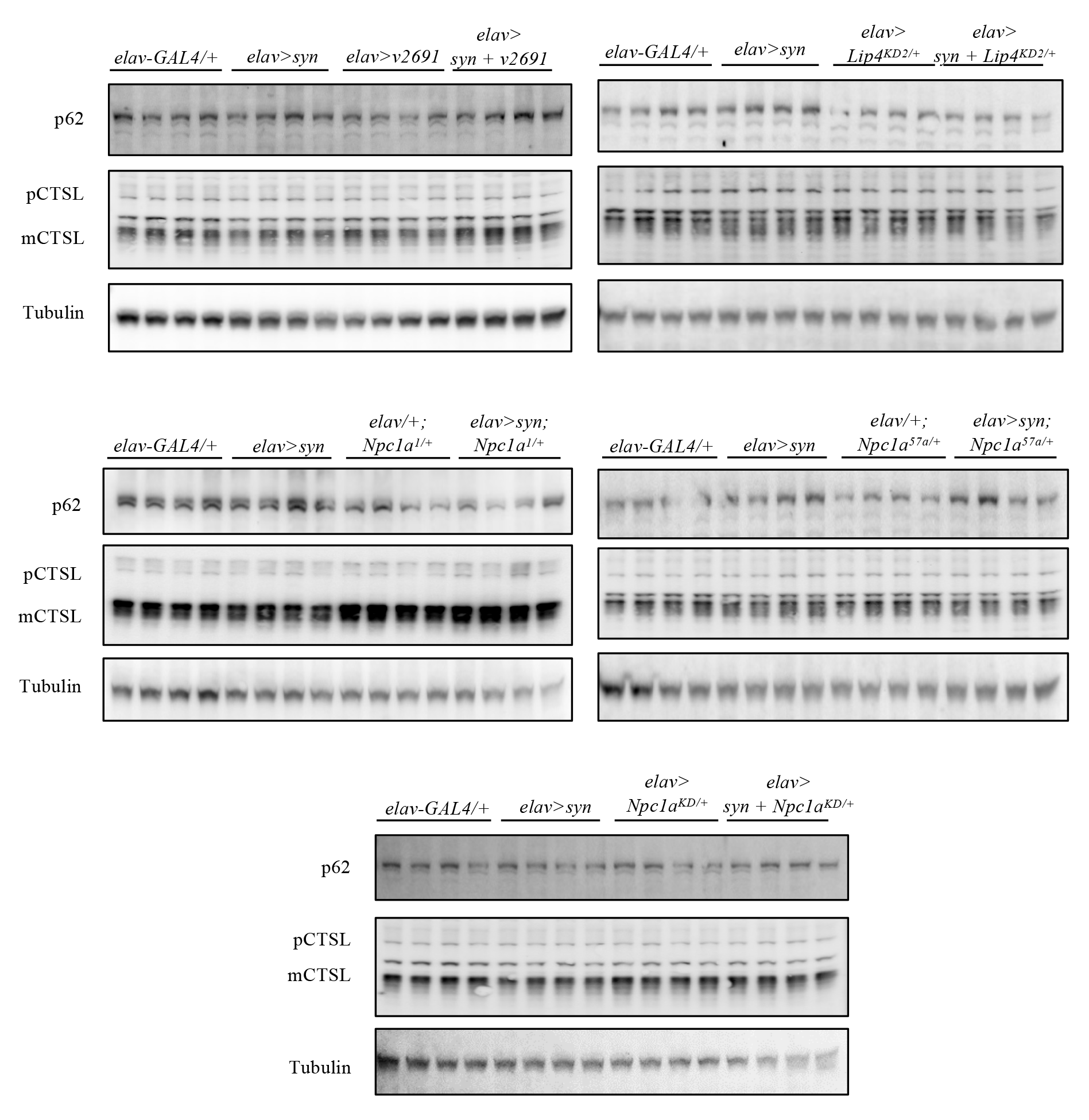

Supplement: S6 Fig — Original western blot data is shown for investigations of markers of lysosomal function, including autophagic flux (p62) or Cathepsin L (CTSL) proteolysis, following Npc1a or Lip4 loss-of function. The following RNAi strains were tested: Npc1a (v105405), Lip4 (KD1: v31021; KD2: v106614). We used the v2691 strain as a non-targeting, scramble RNAi. See S5 Fig for quantitation and statistical analysis of these data. For quantitation, all intensity data was normalized to mean intensity of elav>syn bands on each blot, permitting an integrated analysis. Poorly-transferred p62 bands were excluded from analysis, including all of the elav/+ control lanes on the Npc1a57 blot (middle row, right) and one of the elav>Lip4KD2/+ control lanes (top row, right). (TIF) [file pgen.1010760.s006.tif]

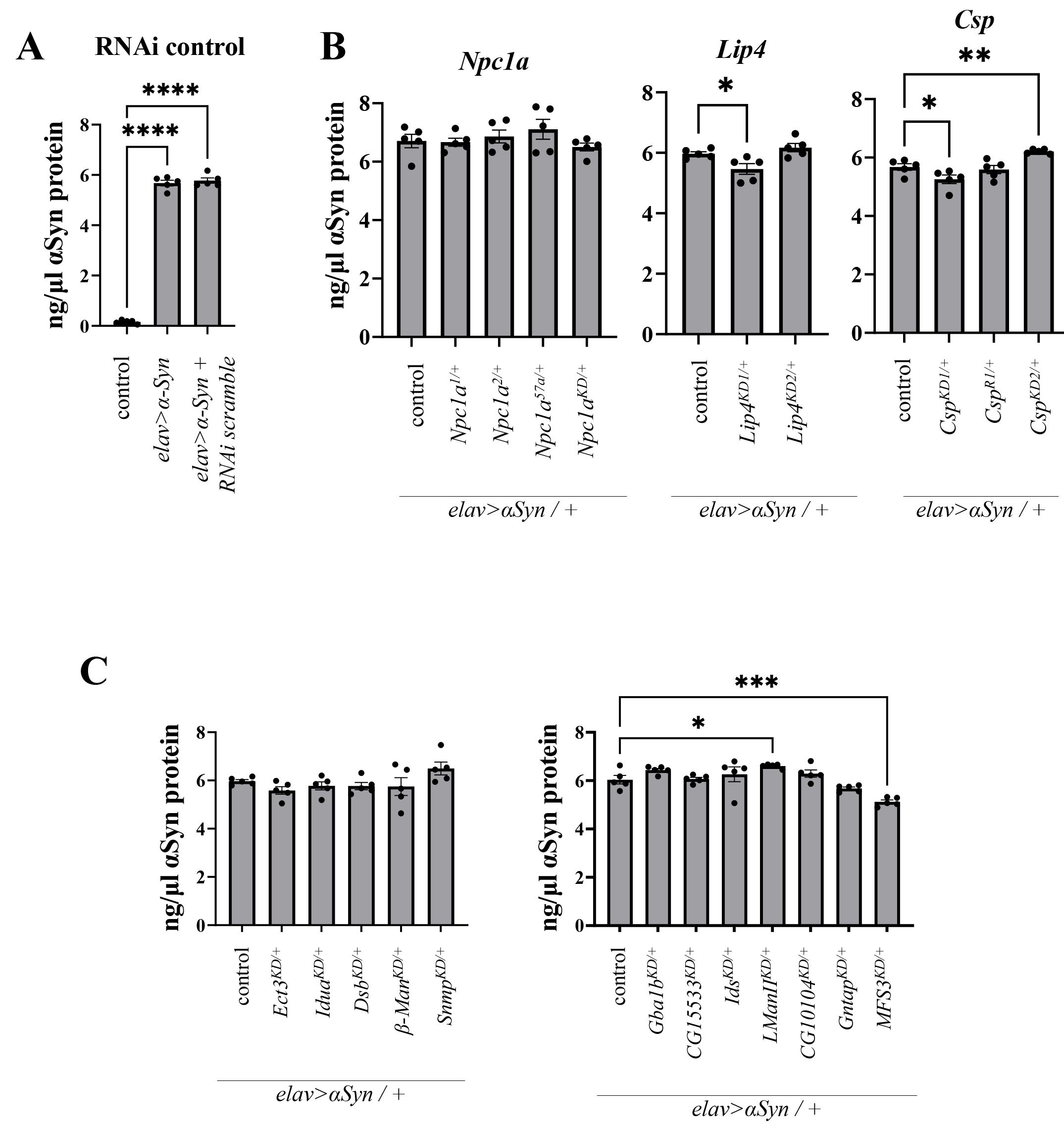

Supplement: S7 Fig — Enzyme linked immunosorbent assays (ELISA) were performed for quantification of total α-synuclein levels from 10-day-old fly head homogenates. α-synuclein was expressed pan-neuronally using the elav-GAL4 driver (elav> αsyn). LSD gene modifiers were manipulated using RNA interference knockdown (RNAi) or loss-of-function alleles; all such manipulations were tested in heterozygosity. (A) α-synuclein protein is sensitively and specifically detected by the ELISA in elav> αsyn flies but not wildtype controls. α-synuclein protein levels are unchanged following co-expression of a non-targeting UAS-RNAi-scramble control construct. (B) Manipulations of either Npc1a, Lip4, or Csp do not cause any consistent changes in α-synuclein protein levels. The following RNAi strains were tested: Npc1a (v105405), Lip4 (KD1: v31021; KD2: v106614), Csp (KD1: 6395R-2, KD2: v103201). (C) For all other LSD gene modifiers, the RNAi transgene generating the strongest locomotor phenotype was selected for ELISAs: Ect3 (3132R-2); Idua (v13244); Dsb (v4100); ß-Man (v15028); Snmp1 (v42496); Gba1b (v101212); CG15533 (v102842); Ids (v105970); LManII (v108218); CG10104 (v108431); Gntap (v109400); MFS3 (v330237). Only knockdown of LManII caused a significant increase in α-synuclein protein levels. Reduction of MFS3 decreased α-synuclein protein levels. All other manipulations did not significantly impact α-synuclein protein levels. Quantification based on analysis of at least n = 5 replicate samples per genotype. Statistical comparisons were made using unpaired t-tests, followed by Dunnett’s post-hoc test. Error bars represent the standard error of the mean. *, p<0.05; **, p<0.01; ****, p<0.0001 (TIF) [file pgen.1010760.s007.tif]

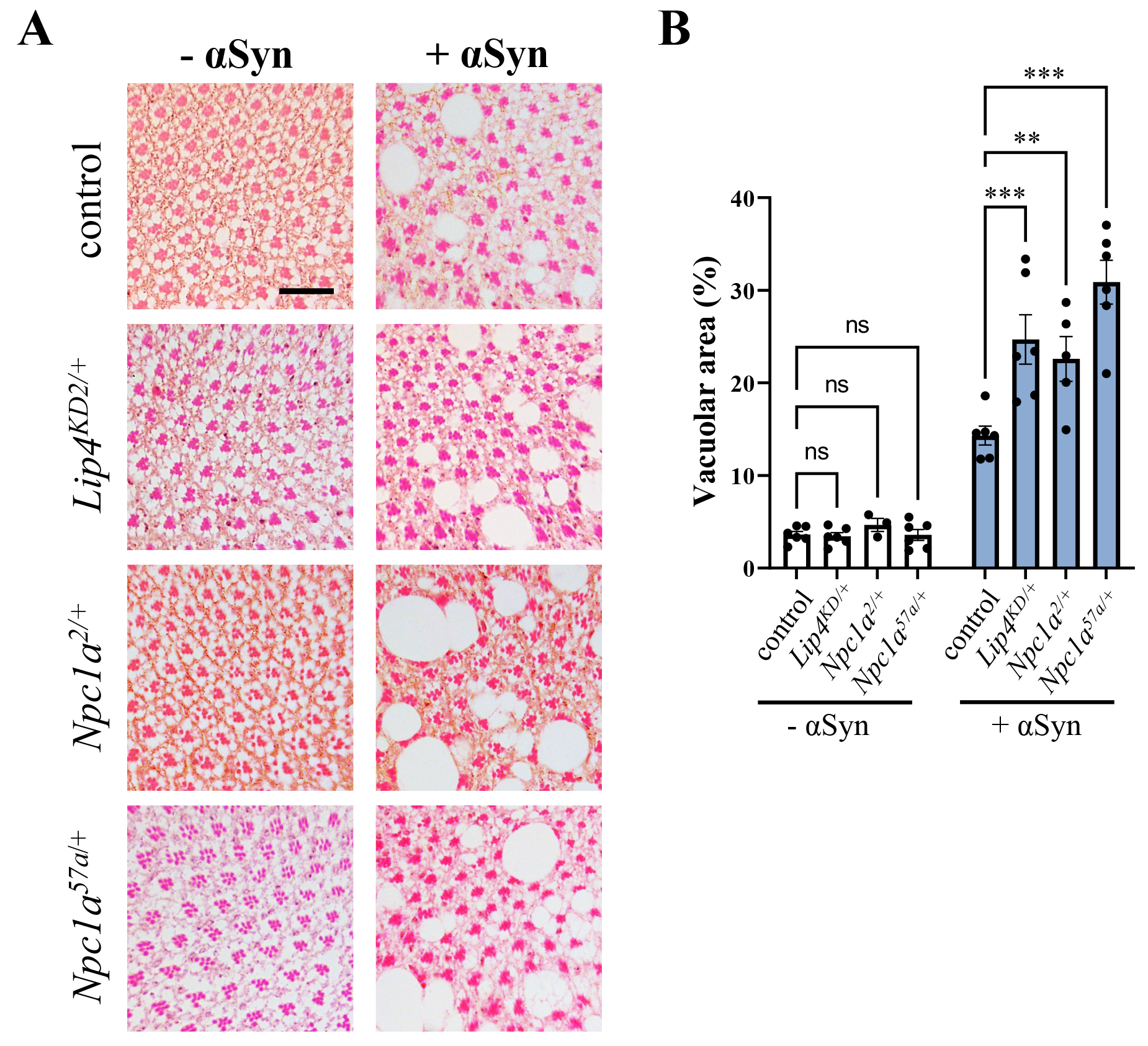

Supplement: S8 Fig — Representative images of retinal histology sections (A) and quantification (B) of additional Npc1a alleles and Lip4 RNAi (v106614), showing consistent enhancement of α-synuclein-mediated neurodegeneration. Quantification based on extent of vacuolar changes (vacuole area / total area) from at least n = 3 animals per genotype. Statistical comparisons were made using unpaired t-tests, followed by Dunnett’s post-hoc test. Error bars represent the standard error of the mean. **, p<0.01; ***, p<0.001; ns, non-significant; Scale bar = 20μm. (TIF) [file pgen.1010760.s008.tif]

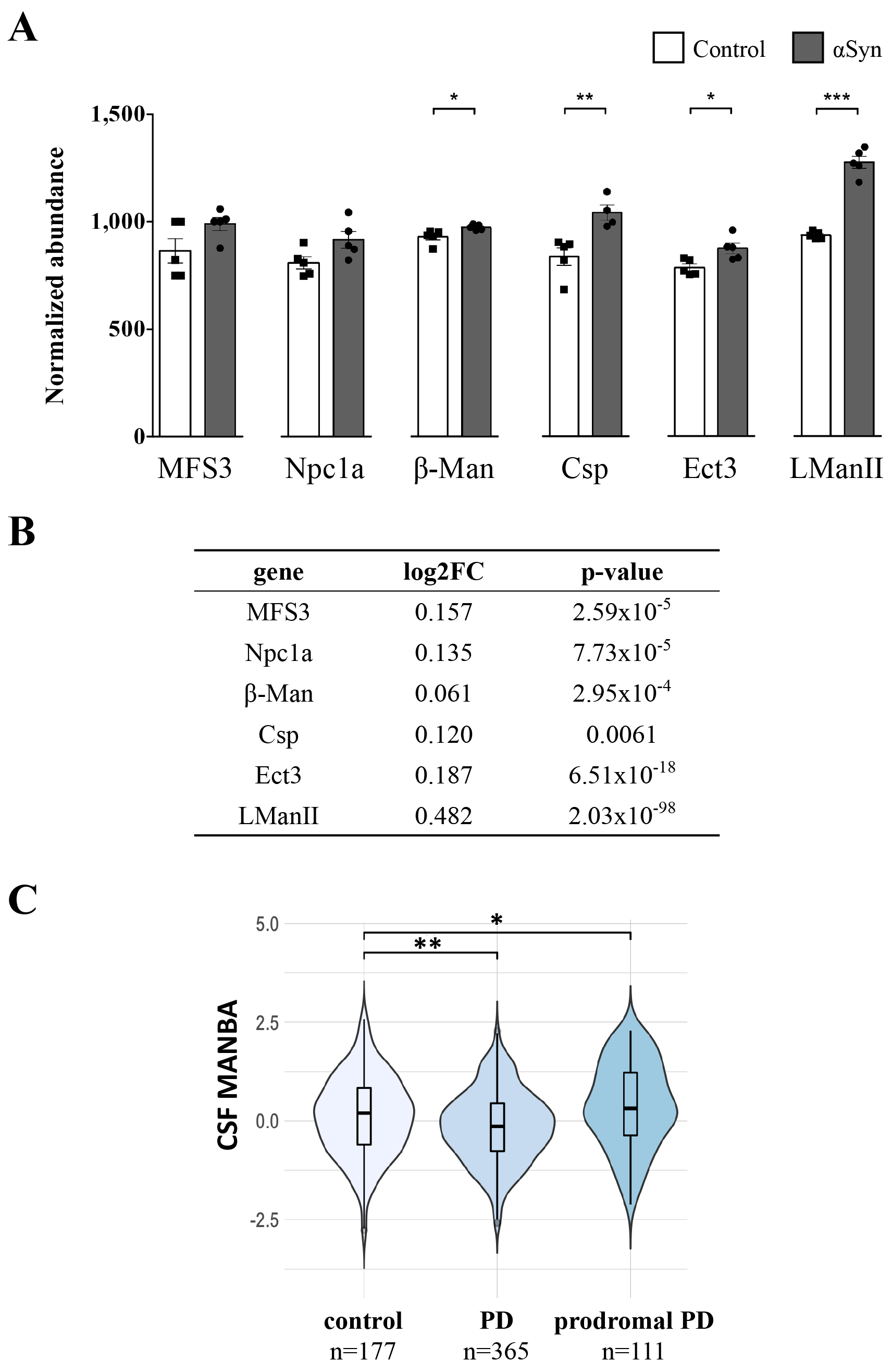

Supplement: S9 Fig — (A, B) Adult fly head homogenates were prepared from elav>αSyn or controls (elav-GAL4 / +), and Tandem Mass Tag proteomics were performed. (A) Cross-sectional comparisons of mean abundance from 10-day-old adults were analyzed using t-tests, considering n = 5 replicate samples for each genotype. Error bars represent the standard error of the mean. *, p<0.05; **, p<0.01; ***, p<0.001. (B) Complementary longitudinal analysis, including 6 aging timepoints between 2 and 21 days. Regression models considered protein expression as an outcome, genotype as a predictor, and included age as a covariate: expression ~ genotype + age. Significance was computed using the Likelihood-ratio test, comparing to the base model: expression ~ age. Benjamini-Hochberg adjusted p-value is shown. log2FC, log base2 (fold-change). (C) MANBA protein levels were examined from human cerebrospinal fluid from the Parkinson’s Progression Markers Initiative (PPMI), including 365 PD cases, 177 controls, and 111 prodromal PD cases, in which early disease biomarkers are present, but clinical manifestations are lacking for the diagnosis. MANBA was significantly elevated in prodromal PD and reduced in clinically manifest PD. (TIF) [file pgen.1010760.s009.tif]
